# Educational and Professional Development Interventions to Strengthen Empathy, Compassion, and Relational Care Among Healthcare Professionals: A Systematic Review

**DOI:** 10.3390/ejihpe16090142

**Published:** 2026-09-17

**Authors:** Alexis Serna-Menor, Juan Pablo Hervás-Pérez, José Antonio Guerra-Guirao, Cibeles Serna-Menor, Gema Mata-González, Andrés García-Notario, María Arnazazu Sánchez-Calabuig, Ivan Herrera-Peco

**Affiliations:** 1 PhD Program in Pharmacy, Faculty of Pharmacy, Complutense University of Madrid, 28040 Madrid, Spain; alexsern@ucm.es (A.S.-M.); ciserna@ucm.es (C.S.-M.); 2 University Center for Health Sciences-HM Hospitals (CUHMED), Camilo Jose Cela University, 28660 Madrid, Spain; gema.mata@ucjc.edu (G.M.-G.); masanchez@ucjc.edu (M.A.S.-C.); 3 Instituto de Investigación Sanitaria HM Hospitales, 28015 Madrid, Spain; 4 Analytical Chemistry Unit, Department of Chemistry in Pharmaceutical Sciences, Faculty of Pharmacy, Complutense University of Madrid, 28040 Madrid, Spain; jphervas@ucm.es; 5 Departamento de Farmacología, Farmacognosia y Botánica, Facultad de Farmacia, Universidad Complutense de Madrid, 28040 Madrid, Spain; jaguerra@ucm.es; 6 Facultad de Ciencias Biomédicas y de la Salud, Universidad Alfonso X el Sabio, Avda Universidad, 1, Villanueva de la Cañada, 28691 Madrid, Spain; apsico@uax.es

**Keywords:** humanization of care, clinical empathy, compassion, patient-centered care, professional–patient relations, communication skills training, continuing professional development, healthcare professionals

## Abstract

Humanized healthcare depends on professionals’ capacity to communicate empathically, respond compassionately, and establish therapeutic relationships. This globally focused systematic review aimed to synthesize comparative evidence on educational and professional development interventions designed to strengthen humanistic and relational practice among patient-facing healthcare professionals across international healthcare contexts. MEDLINE via PubMed, Scopus, Web of Science Core Collection, and the Cochrane Central Register of Controlled Trials (CENTRAL) were searched. Comparative intervention studies involving practicing healthcare professionals were included. Findings were synthesized narratively by relational domain and assessment source. Risk of bias was evaluated using the revised Cochrane risk-of-bias tool for randomized trials (RoB 2), its extension for cluster-randomized trials, and the Risk Of Bias In Non-randomized Studies of Interventions Version 2 (ROBINS-I V2). Thirty-five reports representing 33 independent studies were included. Reports addressed clinical empathy and emotional responsiveness (n = 22); compassion, caring, and humanistic practice (n = 7); and trust, rapport, and therapeutic relationships (n = 6). Favorable effects were most consistent for proximal professional outcomes, including self-reported empathy, compassion, caring competence, confidence, and self-efficacy. Several studies also reported improvements in specific observed communication behaviors. However, patient- and family-reported outcomes were less frequently assessed and showed less consistent favorable between-group differences. Longer-term maintenance was also uncertain. Most individually randomized trials raised some concerns about risk of bias (13/19), while the two cluster-randomized trials were judged as having some concerns and high risk, respectively; all 12 nonrandomized comparative studies were at serious risk of bias. Educational interventions may strengthen selected relational competencies, but evidence of sustained transfer to routine practice, patient experience, trust, and therapeutic alliance remains limited and heterogeneous.

## 1. Introduction

The quality of healthcare depends not only on technical accuracy and clinical effectiveness, but also on patients’ experiences and therapeutic relationships. Humanized healthcare recognizes patients’ clinical, emotional, social, relational, and ethical needs. It encompasses dignity, respect, emotional support, autonomy, shared decision-making, continuity, trust, effective information exchange, and feeling heard and understood. These dimensions characterize person-centered care, caring behaviors, and professional–patient relationships across healthcare settings and populations (Bardaie et al., 2026; Correia et al., 2025; Hansen et al., 2025; Kassa et al., 2025; van Erp et al., 2025). For the purposes of this review, humanized practice was operationalized through relational competencies and observable clinical behaviors that promote dignity, emotional responsiveness, patient participation, and therapeutic connection.

Empathy, compassion, caring competence, communication, and relational competence are interconnected but distinct. Empathy involves understanding patients’ experiences and emotions and responding appropriately (H. L. Ngo et al., 2025), and is expressed through verbal, nonverbal, and interactional behaviors that acknowledge emotions and convey understanding and support (Ta et al., 2026). Compassion entails recognizing and responding to suffering, whereas caring behaviors integrate technical competence with comfort, reassurance, respect, connectedness, and trust (Bardaie et al., 2026; Varghese & Khakha, 2025). Touch may convey reassurance and attunement, although its meaning depends on patient preferences and sociocultural context (Buono et al., 2025). In sensitive situations, these competencies require ethical awareness, clinical knowledge, and the ability to involve patients and families in difficult conversations (Pusa et al., 2024).

Interventions strengthening patient–clinician relationships have produced small favorable effects on physical health, health-related quality of life, and healthcare use, although certainty remains low and heterogeneity substantial (Neal et al., 2026). Individualized communication has been associated with greater participation in self-management, while empathy, trust, continuity, information exchange, and feeling heard relate to more favorable patient experiences (Correia et al., 2025; Iroegbu et al., 2025; Quigley et al., 2024). These experiences are shaped by language, sociocultural factors, and clinical context (Collet et al., 2022; Primeau et al., 2024; Quigley et al., 2025). Nevertheless, shared decision-making remains limited in routine practice (Ubbink et al., 2025).

Humanistic competencies are shaped by personal, professional, and contextual factors rather than representing exclusively stable individual characteristics (Gohri et al., 2025; Pavlova et al., 2022). Their relationship with professional well-being is complex. Mindfulness and supportive organizational conditions may support empathy and well-being, although the evidence remains heterogeneous and organizational compassion is inconsistently defined (Nielsen et al., 2025; Tement et al., 2021; Zhou, 2025). Ethical sensitivity has also been associated with caring behaviors and perceived nursing-care quality (Nazari et al., 2025), supporting interventions that integrate relational and ethical dimensions.

Various educational interventions target these competencies. An umbrella review of 42 reviews associated empathy training with favorable outcomes, particularly with active, experiential, reflective, or patient-involved learning (Edwards et al., 2025). Meta-analyses suggest moderate improvements in cognitive and affective empathy, but methodological and measurement heterogeneity prevents identification of a universally superior approach (H. Ngo et al., 2025; H. L. Ngo et al., 2025). Balint groups may improve empathy, communication, and management of professional–patient relationships, although the evidence has substantial methodological limitations (Gong et al., 2024; Xu et al., 2025). Other strategies include arts-based education, narrative medicine, reflective writing, emotional intelligence training, simulation, role-play, mindfulness, virtual reality, and distance learning (Maity et al., 2025; Mojarrad et al., 2026; Pira et al., 2025; Rigatos et al., 2025). Together, these findings support their modifiability but also reveal considerable variation in intervention mechanisms, intensity, and clinical delivery context.

Communication programs combine didactic instruction, role-play, feedback, reflection, and group discussion and report favorable changes in communication behaviors, confidence, and self-efficacy (Peimani et al., 2025). Patient- and family-centered programs may improve nurses’ perceived competence, but inconsistently address collaboration, self-awareness, and professional values (Joo et al., 2025). Training and technology also target caring competence, patient-centered practice, and shared decision-making (Bollos et al., 2023; Ubbink et al., 2025). Nevertheless, improved knowledge, confidence, or perceived competence does not demonstrate better clinical communication. Interventions targeting patients and clinicians may improve patient-centered communication, but effects on care and patient-related outcomes are less consistent (McDarby et al., 2025).

Interpretation depends on outcome assessment. Self-reported empathy, confidence, self-efficacy, and caring competence reflect perceived abilities, not demonstrated performance or behavioral change. Confidence-based instruments should not substitute for performance measures (Jagemann et al., 2025). Observational and patient-reported communication instruments vary extensively, limiting comparison and synthesis (Al Sulaimi et al., 2026), while measurement-based approaches may overlook the contextual complexity of clinical experience (Dey et al., 2025). Patient-reported experience, observation, recorded interactions, and external assessment provide complementary evidence of transfer into practice (Bardaie et al., 2026; Quigley et al., 2024; Ta et al., 2026). Reviews of arts- and technology-supported empathy education have identified small samples, heterogeneous instruments, predominantly pre–post designs, and limited long-term follow-up (Mojarrad et al., 2026; Pira et al., 2025). Immediate self-reported improvement must therefore be distinguished from sustained change in observable behavior and patient experience. For the purposes of this review, humanized practice was operationalized as a multidimensional relational construct expressed through professional competencies, observable clinical behaviors, and patient-oriented relational processes that promote dignity, emotional responsiveness, participation, and therapeutic connection. This operationalization was intended to integrate the relational dimensions described above rather than to propose a new theoretical definition of humanized care. The three analytical domains were defined as follows: (1) clinical empathy and emotional responsiveness, encompassing understanding of patients’ experiences and emotions, recognition of emotional cues, perspective-taking, and empathic responding; (2) compassion, caring, and humanistic practice, encompassing responses to suffering, caring attitudes and behaviors, dignity, emotional support, and person-centered caring; and (3) trust, rapport, and therapeutic relationships, encompassing trust-building, rapport, therapeutic or working alliance, autonomy support, shared decision-making, and broader professional–patient relationship processes. These domains were used as an organizing framework for the synthesis rather than as mutually exclusive theoretical constructs because some interventions and outcomes spanned more than one relational dimension. Knowledge, confidence, and self-efficacy were considered proximal educational outcomes rather than direct evidence of improved relational care.Previous reviews have generally focused on individual constructs, professional groups, educational modalities, or clinical settings (Edwards et al., 2025; Joo et al., 2025; McDarby et al., 2025; H. Ngo et al., 2025; H. L. Ngo et al., 2025). Although this literature has established that empathy, communication, compassion, and related competencies can be modified through education, it does not provide an integrated account of how these interventions perform across related domains of humanistic and relational care. Previous syntheses have not consistently distinguished whether favorable findings reflect proximal professional perceptions, observable relational behavior, or patient- and family-reported experience, nor whether these changes persist beyond the immediate educational setting.

The added value of the present review therefore lies not simply in combining three relational domains, but in examining them within a common analytical framework restricted to comparative interventions involving practicing, patient-facing healthcare professionals. The review integrates clinical empathy and emotional responsiveness; compassion, caring, and humanistic practice; and trust, rapport, and therapeutic relationships, while simultaneously distinguishing outcome assessment sources, examining persistence over time, and evaluating whether proximal educational gains translate into observable clinical behavior or patient-related outcomes.

Accordingly, this globally focused systematic review aimed to synthesize comparative evidence on educational and professional development interventions targeting these three domains among patient-facing healthcare professionals across countries and healthcare systems. It also examined intervention characteristics, assessment sources, follow-up periods, risk of bias, and evidence of transfer from proximal educational outcomes to relational clinical practice and patient experience.

## 2. Materials and Methods

### 2.1. Review Design and Registration

This systematic review was conducted according to a predefined protocol and is reported in accordance with the Preferred Reporting Items for Systematic Reviews and Meta-Analyses 2020 statement (PRISMA 2020) (Page et al., 2021). The narrative synthesis was structured following the Synthesis Without Meta-analysis reporting guideline (SWiM) (Campbell et al., 2020). The protocol was registered in the International Prospective Register of Systematic Reviews (PROSPERO; CRD420261456794).

### 2.2. Eligibility Criteria

Eligibility criteria were defined according to population, intervention, comparator, outcomes, study design, and publication characteristics.

Studies were eligible when participants were healthcare professionals with direct patient-care responsibilities, including physicians, nurses, pharmacists, psychologists, physiotherapists, occupational therapists, and other allied healthcare professionals. Residents and postgraduate specialty trainees were eligible when they had direct clinical responsibilities. Studies involving mixed samples were retained when data for eligible professionals could be extracted separately or when healthcare professionals with direct patient-care responsibilities represented more than 50% of the total participant sample. Undergraduate students without clinical-care responsibilities and studies exclusively involving patients, family members, informal caregivers, nonclinical personnel, or administrative staff were excluded.

Eligible interventions were structured educational, behavioral, or professional development programs explicitly intended to strengthen humanistic, relational, compassionate, person-centered, or patient-centered care. Relevant intervention targets included empathy, emotional responsiveness, compassion, caring behaviors, communication, active listening, perspective-taking, shared decision-making, therapeutic relationships, trust, rapport, and other observable relational competencies. Face-to-face, online, blended, simulation-based, coaching, mentoring, and workplace-based formats were eligible. Programs primarily addressing mindfulness, self-compassion, resilience, stress, burnout, or professional well-being were included only when they contained an explicit patient-oriented relational component and reported at least one eligible relational, behavioral, or patient-related outcome.

Eligible comparators included usual practice or training, no intervention, waiting-list conditions, attention controls, alternative educational interventions, or comparisons between two eligible training approaches. A concurrent comparator was required.

Individually randomized trials, cluster-randomized trials, nonrandomized controlled intervention studies, and controlled before-and-after studies were eligible. Uncontrolled before-and-after studies, single-group pilot or feasibility studies, post-intervention-only evaluations, and studies using exclusively historical or external comparison groups were excluded. Only full-text articles published in peer-reviewed journals and written in English were eligible. No restrictions were imposed on publication date, geographical location, healthcare setting, or professional discipline. Conference abstracts, protocols, theses, dissertations, preprints, editorials, commentaries, and publications without evaluable outcome data were excluded. No restrictions were imposed on publication date, country, geographic region, or healthcare system.

### 2.3. Information Sources and Search Strategy

Systematic searches were conducted on 22 July 2026 in MEDLINE via PubMed, Scopus, Web of Science Core Collection, and the Cochrane Central Register of Controlled Trials (CENTRAL). These databases were selected to provide complementary coverage of biomedical, multidisciplinary, citation-indexed, and controlled-intervention literature across the range of healthcare professions considered in the review. Because the review was designed as a cross-disciplinary synthesis rather than a profession-specific review, the search strategy prioritized broad databases covering medicine, nursing, allied health, and other patient-facing disciplines. CINAHL was not included in the original database search.

The search strategy combined four conceptual blocks: healthcare professionals; structured educational or professional development interventions; humanistic, relational, compassionate, or person-centered care; and comparative or controlled intervention designs. Search terms included controlled vocabulary and free-text alternatives for healthcare professionals, training, continuing education, professional development, empathy, compassion, humanized care, caring behaviors, patient-centered or person-centered care, communication, therapeutic relationships, trust, rapport, shared decision-making, and controlled studies. Syntax, field tags, truncation, and proximity operators were adapted to each database.

Searches were limited to full-text journal articles published in English, with no restrictions on publication date. Database-specific limits were applied according to the available indexing and search functions of each platform. The eligibility restrictions described above were implemented using database-specific filters where available. PubMed was limited to human studies and eligible article types; Scopus and Web of Science Core Collection were restricted to articles; and CENTRAL was searched within the Trials collection. No restrictions by source, subject category, or open-access status were applied. The complete database-specific search strategies, including the exact syntax, field tags, truncation operators, limits, search date, and number of records retrieved, are provided in Supplementary Table S1.

### 2.4. Screening and Elegibility Assesment

All retrieved records were combined in a single screening database, and duplicates were removed before eligibility assessment. Study selection was conducted in two stages. First, titles and abstracts were screened against the eligibility criteria. Records were retained for full-text assessment whenever eligibility was uncertain, or the available information was insufficient for a reliable decision. Second, the full texts of potentially eligible reports were assessed using the complete population, intervention, comparator, outcome, and study-design criteria.

Title and abstract screening and full-text eligibility assessment were conducted independently by two reviewers (A.S.-M. and J.P.H.-P.). Disagreements were resolved through discussion and consensus. When consensus could not be reached, a third reviewer (I.H.-P.) acted as arbiter. Reasons for exclusion were recorded at the full-text stage. Reports that could not be obtained after reasonable retrieval efforts were classified as not retrieved.

Multiple publications arising from the same study were linked using author names, setting, participant characteristics, intervention details, recruitment dates, sample size, and trial registration information. Companion publications were considered jointly and counted as a single independent study, while all reports contributing eligible information were retained in the review. The study-selection process is presented in the PRISMA 2020 flow diagram.

### 2.5. Data Extraction

Data were extracted using a standardized form that was pilot-tested before use. Two reviewers (A.S.-M. and J.P.H.-P.) independently extracted the data. Discrepancies were resolved through consensus, with consultation of a third reviewer (I.H.-P.) when necessary. Study authors were not contacted to obtain or verify missing or unclear information; data extraction and interpretation were based on the information available in the published reports and associated materials.

The extracted information included bibliographic details; country and healthcare setting; study design; recruitment procedures; professional group; sample size and participant characteristics; intervention theory or framework; intervention components; delivery format; intervention provider; duration, intensity, and frequency; comparator characteristics; outcome constructs and measurement instruments; source of outcome assessment; assessment time points; attrition; and findings for the outcomes relevant to this review. Between-group estimates, 95% confidence intervals, effect sizes, and *p*-values were extracted when reported. Adverse or unintended effects, funding sources, and conflicts of interest were also recorded when available.

For studies reported in multiple publications, data were initially extracted at the report level and subsequently integrated at the study level. Companion reports were identified by comparing authorship, participant characteristics, recruitment settings and periods, intervention characteristics, and sample sizes. When information differed across companion reports, data were obtained from the most complete report or from the report corresponding to the relevant outcome or follow-up assessment. Reports from the same underlying study were not counted as independent studies.

Between-group comparisons were prioritized over within-group changes. When multiple estimates were reported, prespecified adjusted estimates were prioritized over unadjusted estimates when methodologically appropriate. For cluster-randomized studies, estimates accounting for the clustered design were prioritized. No numerical values were imputed when data were unavailable.

### 2.6. Outcomes and Analytical Framework

The primary outcomes were patient-oriented relational competencies, observable professional relational behaviors, and patient- or family-reported relational experiences. To organize the heterogeneous outcomes included in the review, we used a review-level analytical framework comprising three conceptually related domains. The framework was developed for the purposes of the synthesis from the relational constructs specified in the eligibility criteria and was not intended to represent mutually exclusive theoretical categories.

Clinical empathy and emotional responsiveness, including cognitive and affective empathy, perspective-taking, recognition of emotional cues, empathic communication, and responses to patients’ emotions.

Compassion, caring, and humanistic practice, including compassion competence, caring attitudes and behaviors, humanistic-practice ability, dignity, emotional support, and person-centered caring.

Trust, rapport, and therapeutic relationships, including therapeutic alliance, working alliance, trust-building, rapport, autonomy support, shared decision-making, and broader professional–patient relationship measures. Each report was assigned to one primary relational domain for study-level classification and counting. When outcomes spanned more than one domain, the primary domain was determined according to the intervention’s principal relational target and the principal eligible relational outcome or outcomes reported in the study. Outcomes relevant to additional domains were retained in the corresponding narrative synthesis but were not used to count the report more than once.

Overlapping constructs were classified according to their operational definition and measurement rather than their terminology alone. Relational presence was therefore not treated as a separate domain; depending on how it was operationalized, presence-related outcomes were classified within the compassion/caring or trust/therapeutic-relationship domains.

### 2.7. Risk-of-Bias Assessment

Risk of bias was assessed using design-specific Cochrane tools. Individually randomized trials were evaluated using the revised Cochrane risk-of-bias tool for randomized trials (RoB 2) (Sterne et al., 2019). Cluster-randomized trials were assessed using the RoB 2 extension for cluster-randomized designs. Nonrandomized intervention studies were evaluated using the Risk Of Bias in Non-randomized Studies of Interventions tool (ROBINS-I) (Sterne et al., 2025).

For individually randomized trials, the assessment considered bias arising from the randomization process, deviations from intended interventions, missing outcome data, outcome measurement, and selection of the reported result. For cluster-randomized trials, additional consideration was given to the timing of participant identification and recruitment relative to cluster allocation, baseline imbalances, the number of randomized clusters, and whether the analysis appropriately accounted for clustering. ROBINS-I assessments considered confounding, participant selection, intervention classification, deviations from intended interventions, missing data, outcome measurement, and selective reporting.

Risk-of-bias assessments were performed at the outcome/result level rather than as a single study-wide judgment. When an eligible relational outcome was explicitly identified as primary in the original study, that outcome was selected for assessment. When no eligible relational outcome was explicitly designated as primary, the outcome most directly aligned with the intervention’s principal relational target and the study’s primary relational domain was selected. When a study contributed substantively to the synthesis through eligible relational outcomes representing different assessment sources or distinct relational domains, more than one outcome was assessed separately. Knowledge, confidence, self-efficacy, and other proximal educational outcomes were not treated as principal relational outcomes for this purpose. Where several results were available for the same selected outcome, the result or results contributing to the main comparative synthesis were used for the risk-of-bias assessment. Attention was given to whether self-reported outcomes could have been influenced by participants’ knowledge of intervention allocation and whether observer- or patient-reported assessments were conducted independently of the intervention team.

Assessments were conducted independently by two reviewers, with disagreements resolved through consensus and, when necessary, arbitration by a third reviewer. Domain-level judgments and supporting justifications are presented in Supplementary Tables S5–S7. Risk-of-bias judgments were not converted into numerical quality scores, and studies were not excluded from the synthesis solely because of their risk-of-bias classification.

### 2.8. Data Synthesis

A random-effects meta-analysis was planned when studies were sufficiently comparable in population, intervention, comparator, outcome construct, measurement method, and assessment time. However, the included evidence showed substantial clinical and methodological heterogeneity across professional groups, intervention content and intensity, comparator conditions, measurement instruments, assessment sources, follow-up periods, and study designs. Consequently, statistical pooling was not considered methodologically appropriate.

A structured narrative synthesis was therefore conducted following SWiM recommendations (Campbell et al., 2020). Studies were grouped according to relational domain, source of outcome assessment, study design, professional group, intervention format, comparator, and follow-up period. Within each group, the synthesis considered the direction and magnitude of between-group effects, precision when reported, consistency across studies and assessment sources, persistence over time, and risk of bias. Conclusions were not based solely on the number of statistically significant findings.

Results from companion publications were integrated at the study level to avoid double counting. Attention was given to the progression from proximal educational outcomes, such as knowledge, confidence, or self-efficacy, to observable clinical behavior and patient- or family-reported relational experience.

The protocol anticipated subgroup analyses according to profession, intervention format, outcome type, study design, and follow-up duration, together with sensitivity analyses based on methodological quality. The number and heterogeneity of studies within potentially comparable groups did not support formal subgroup testing or quantitative sensitivity analyses. These characteristics were therefore examined descriptively. Statistical heterogeneity, publication bias, and small-study effects were not calculated because no meta-analysis was performed.

Formal Grading of Recommendations Assessment, Development and Evaluation (GRADE) ratings were not assigned because the included evidence could not be organized into sufficiently homogeneous, discrete, and clinically coherent intervention–comparison–outcome bodies of evidence. Rather, the included studies could not be organized into sufficiently discrete and clinically coherent intervention–comparison–outcome bodies without combining substantially different relational constructs, intervention approaches, comparators, assessment sources, measurement instruments, and follow-up periods. In particular, the three relational domains used in this review were intended as broad analytical categories rather than as single homogeneous outcomes. Assigning a single certainty rating to these domains would therefore have risked implying a level of comparability that was not supported by the underlying evidence. Confidence in the narrative findings was instead appraised descriptively by considering risk of bias, consistency, directness, precision, assessment source, transfer to routine clinical practice, and persistence of effects.

## 3. Results

### 3.1. Study Selection

**Figure 1:**
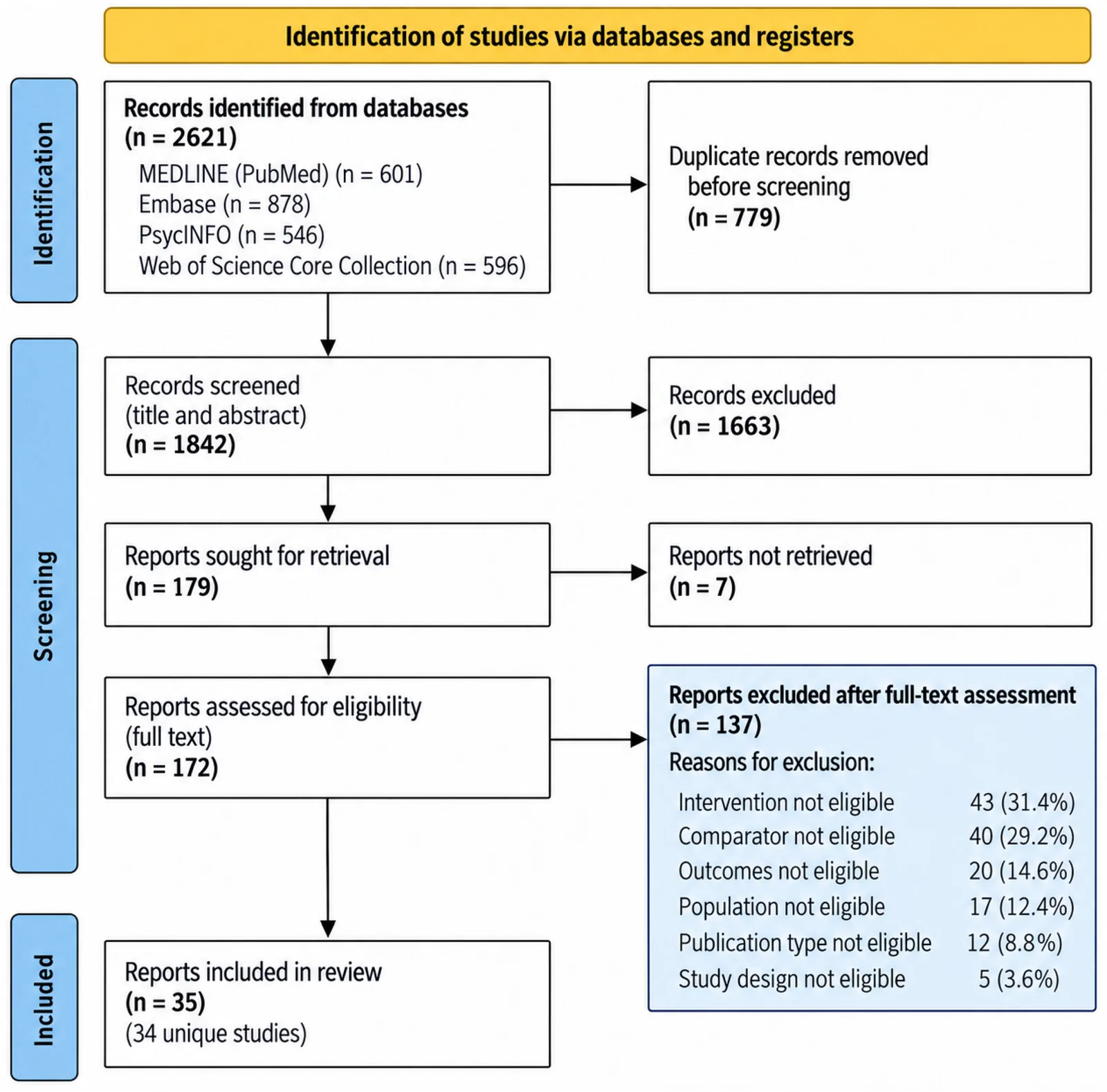
PRISMA 2020 flow diagram of study identification, screening, eligibility assessment, and inclusion.

The database searches identified 2621 records: 982 from Scopus, 720 from MEDLINE/PubMed, 653 from Cochrane CENTRAL, and 266 from Web of Science. After 779 duplicates were removed, 1842 unique records underwent title and abstract screening, of which 1663 were excluded. A total of 179 reports were sought for retrieval; seven could not be retrieved, and 172 were assessed for eligibility. Following full-text assessment, 137 reports were excluded; the most frequent reasons were an ineligible intervention (n = 43; 31.4%) and an ineligible comparator (n = 40; 29.2%), followed by ineligible outcomes (n = 20; 14.6%), population (n = 17; 12.4%), publication type (n = 12; 8.8%), and study design (n = 5; 3.6%). Thirty-five reports, corresponding to 33 independent studies, were included in the review. The complete study-selection process, including the reasons and corresponding numbers for full-text exclusion, is presented in Figure 1. Finally, 35 reports, corresponding to 33 independent studies, were included in the review. Two study families were represented by companion publications: Gibon et al. (2013) and Merckaert et al. (2015), and Fallowfield et al. (2002, 2003). Companion reports were linked and treated as single studies in study-level analyses. The study-selection process and the verified reasons for full-text exclusion are presented in Figure 1.

### 3.2. Characteristics of the Included Evidence

The 35 reports, representing 33 independent studies, were published between 1995 and 2026 and covered diverse clinical and educational settings. At the study level, nurses were the most frequently represented professional group (n = 14; 42.4%), followed by physicians or residents (n = 12; 36.4%), multidisciplinary or mixed professional samples (n = 4; 12.1%), other patient-facing healthcare professional samples (n = 2; 6.1%), and physiotherapists (n = 1; 3.0%). Oncology or cancer care was the most frequently represented clinical context (n = 10; 30.3%), followed by general hospital or non-specialty settings (n = 8; 24.2%) and primary care or general practice (n = 4; 12.1%). HIV care, long-term or older-adult care, and pediatric or adolescent care were each represented in two studies (6.1%), whereas rehabilitation, hemodialysis, critical care, ophthalmology, and obstetrics were each represented in one study (3.0%). Sample sizes ranged from 12 clinicians in a pilot trial to more than 3000 trained and untrained physicians in a controlled institutional evaluation (Table 1).

At the report level, 22 reports (62.9%) were primarily classified under clinical empathy and emotional responsiveness, seven (20.0%) under compassion, caring, and humanistic practice, and six (17.1%) under trust, rapport, and therapeutic relationships. Because presence was operationalized heterogeneously across studies, as warmth, involvement, empathic attention, supportive behavior, caring interaction, rapport, or working alliance, it was not treated as a standalone relational domain. Presence-related outcomes were instead classified according to their operational definition within either the compassion/caring or trust/therapeutic-relationship domains (Table 1).

For descriptive purposes, each independent study was also classified according to its dominant intervention approach, recognizing that many programs incorporated more than one educational component. Communication- or relationship-focused workshops and curricula were the most common approach (n = 18; 54.5%), followed by interventions primarily based on simulation, role-play, or standardized-patient methods (n = 6; 18.2%) and motivational interviewing (n = 3; 9.1%). Narrative medicine or storytelling represented the dominant approach in two studies (n = 2; 6.1%). One study each (3.0%) was primarily based on video interaction analysis, training combined with mentoring, a neuroscience-informed empathy curriculum, or a Watson Human Caring-based intervention. Several programs also incorporated explicit communication or behavioral frameworks, including AIDET and self-determination theory. Exposure ranged from a single brief session to programs lasting several months. Comparators included no training, usual education, wait-list control, video or conventional teaching, alternative educational formats, and lower-intensity active interventions. Consequently, several studies estimated the incremental effect of one educational approach over another rather than the effect of training versus no intervention (Table 1).

### 3.3. Outcome Measurement and Analytical Grouping

Outcome measurement varied substantially across the included studies, and no single instrument predominated across the evidence base. The Consultation and Relational Empathy (CARE) measure was used in two independent studies, and variants of the Jefferson Scale of Empathy were also used in two studies. The remaining named instruments were generally used in single studies. Table 2 groups the principal instruments and assessment approaches according to the relational construct and primary analytical domain they assessed.

Instruments were also classified according to the source of the outcome assessment. Professional self-report measures assessed perceived empathy, compassion, caring competence, humanistic-practice ability, and patient-centered care. Standardized educational and observer-based assessments evaluated demonstrated communication behavior, emotional responsiveness, warmth, involvement, supportive communication, and need-supportive behavior. Patient- or family-reported measures assessed experienced empathy, trust, satisfaction, communication, and broader relational experience. Knowledge, confidence, and self-efficacy were classified as proximal educational outcomes rather than direct measures of relational care. These assessment sources were analyzed separately because perceived competence, observable behavior, and relational care experienced by patients or families are not interchangeable.

### 3.4. Clinical Empathy and Emotional Responsiveness

Clinical empathy and emotional responsiveness constituted the largest evidence domain, with 22 reports. Interventions ranged from curricula explicitly labeled as empathy training to broader relational or communication programs that assessed empathic responding, emotional cue recognition, warmth, affective communication, or patient-perceived empathy. The principal constructs, instruments, and assessment sources are summarized in Table 2, while the study-level findings are presented in Supplementary Table S2. Findings are discussed separately below for professional or standardized educational assessments, observer-rated behavior, and patient- or family-reported outcomes.

#### 3.4.1. Professional and Standardized Educational Assessments

Liang et al. (2025) reported higher post-training scores on the Set the Stage, Elicit Information, Give Information, Understand the Patient’s Perspective, and End the Encounter (SEGUE) framework, the Consultation and Relational Empathy (CARE) measure, and the Chinese Physician–Patient Communication Assessment Scale (CPPC-AS) after situational simulation combined with the Acknowledge, Introduce, Duration, Explanation, and Thank You (AIDET) framework than after traditional teaching. Hussien et al. (2025a) reported greater post-training professional self-reported empathy following standardized-patient compassion training. Du et al. (2025) found higher narrative competence and objective structured clinical examination (OSCE) performance after case-based narrative medicine education. Serrada-Tejeda et al. (2026), however, found no between-group difference in Interpersonal Reactivity Index scores when an aging-simulation suit was added to education received by both groups.

#### 3.4.2. Observer-Rated Empathic and Emotion-Responsive Behavior

Observer-coded studies reported between-group differences in specific relational behaviors, although the coding systems and clinical tasks varied substantially. The radiotherapy-team intervention increased assessment, empathy, negotiation, emotional language, and team-resource content during simulated encounters (Gibon et al., 2013), while its companion report identified favorable differences in several supportive behaviors during routine planning sessions (Merckaert et al., 2015). Liénard et al. (2010) reported more assessment and supportive utterances during clinical rounds. Razavi et al. (2002) found a broader repertoire of emotional language, particularly during simulated encounters, whereas differences in routine interviews were less consistent. Kruijver et al. (2001) and Caris-Verhallen et al. (2000) reported favorable differences in affective communication, involvement, warmth, and open questioning. Butow et al. (2008) observed more environment-creating behavior and fewer blocking responses, although several between-group contrasts were imprecise or not statistically significant. Pollak et al. (2016) also reported favorable between-group differences in observer-rated empathy and motivational interviewing behaviors. Although greater observed empathy was associated with higher patient motivation, this relationship was observational and was not interpreted as an intervention effect.

Longer-term maintenance was inconsistent. In the Cancer Research UK trial, the intervention improved several observed communication behaviors during real consultations at the primary assessment (Fallowfield et al., 2002). At 12 months, some trained behaviors remained evident, but expressions of empathy had declined relative to the initial post-training assessment (Fallowfield et al., 2003). These publications were treated as companion reports from the same trial.

#### 3.4.3. Patient- or Family-Reported Empathy and Relational Experience

Patient- and family-reported outcomes were assessed less frequently than professional or observer-rated outcomes. Riess et al. (2012) reported a favorable between-group difference in change in patient-rated CARE scores. Boissy et al. (2016) found modestly higher adjusted patient-experience scores among trained physicians, together with increased physician self-reported empathy. Smith et al. (1995) reported more favorable patient satisfaction and psychosocial-interaction ratings among patients treated by intensively trained residents. Overall, patient- and family-reported findings were less frequent and less consistent than proximal professional or observer-rated outcomes.

### 3.5. Compassion, Caring, and Humanistic Practice

Seven reports evaluated compassion, caring, or humanistic-practice outcomes as their primary relational domain. The evidence was concentrated in nursing and included simulation, storytelling, mentoring, psychological-support training, ethics and therapeutic-communication education, and an intervention based on Watson’s Human Caring framework. Outcomes included professional knowledge, attitudes, self-efficacy, compassion competence, caring behaviors, and humanistic-practice ability (Table S3).

Mau et al. (2025) reported between-group changes in caring knowledge, attitudes, and actions following training and mentoring. Park et al. (2021) found greater changes in perceived patient-centered care and compassion after storytelling and patient-role enactment. Hussien et al. (2025b) reported favorable post-training differences in caregiving behavior, self-efficacy, and compassion competence after simulation-based compassionate-care training. In the cluster-randomized trial by Antonini et al. (2021), the Watson-based intervention produced favorable differences in selected humanistic caring attitudes and behaviors, although the findings varied across dimensions of the 70-item Caring Nurse–Patient Interaction Scale (EIIP-70) and assessment times.

Zhuang et al. (2025) reported higher post-intervention humanistic-practice ability among newly recruited ophthalmic nurses after visual-impairment simulation based on the Data–Information–Knowledge–Wisdom (DIKW) framework. Shimran and Ali (2025) found favorable differences in knowledge and observed practice after an integrated ethics and therapeutic-communication program. Tai and Lin (2026) reported favorable group-by-time differences in nurse competencies. Ward-level patient satisfaction and quality-of-life outcomes favored the intervention wards at 3 months, but these differences were not maintained at 6 months. Because these outcomes were reported as ward-level aggregates, they were interpreted at the ward rather than the individual-patient level.

### 3.6. Trust, Rapport, and Therapeutic Relationships

Dong et al. (2026) reported a favorable immediate between-group difference in skills for addressing medical mistrust, but this difference was not maintained at 6 months. Selected role-play strategies, including autonomy support and checking understanding, showed larger short-term differences. Thom (2000) found favorable differences in most targeted trust-building behaviors, but no clear improvement in global patient trust. Meystre et al. (2013) found no intervention effect on working alliance. The associations observed between individual communication behaviors and alliance strength were observational and were therefore not interpreted as intervention effects (Table S4).

Norfolk et al. (2009) reported favorable differences in rapport-related knowledge, attitudes, and confidence, whereas behavioral transfer was less consistent. Murray et al. (2015) reported a favorable observer-rated difference in need-supportive behavior after self-determination-theory-based communication training. Smith et al. (1995) reported more favorable patient evaluations of psychosocial interactions after intensive resident training. Overall, proximal trust-building or rapport-related skills improved more consistently than global trust or working-alliance outcomes.

### 3.7. Consistency Across Assessment Sources and Persistence of Effects

Patient- or family-reported outcomes were less frequently assessed and showed less consistent favorable between-group differences across studies. Observer-rated behavioral outcomes also improved in multiple trials, particularly for emotional cue recognition, supportive utterances, warmth, involvement, and need-supportive communication. Patient- or family-reported outcomes were less frequently assessed and showed smaller or less consistent between-group differences. Longer-term follow-up was available in a limited subset of studies. Some trained behaviors persisted, whereas empathy expressions, mistrust-related skills, and ward-level patient outcomes were not consistently maintained at later assessments.

### 3.8. Proximal Educational, Complementary, and Unintended Outcomes

Several studies assessed outcomes extending beyond the primary relational constructs. These included professional confidence, self-efficacy, communication knowledge and attitudes, psychosocial orientation, job stress, burnout, quality of working life, patient satisfaction, and perceived quality of care. Because these outcomes were secondary to the purpose of the review, they were interpreted as complementary indicators of professional or organizational impact rather than direct evidence of improved humanistic-relational practice.

Boissy et al. (2016) reported post-training improvements in physician self-efficacy and burnout-related outcomes, including emotional exhaustion, depersonalization, and personal accomplishment. Lower depersonalization and greater personal accomplishment were maintained at the 3-month assessment. Tai and Lin (2026) found a greater reduction in nurses’ perceived job stress in intervention wards at 3 months, although the between-group difference was no longer evident at 6 months. In the cluster-randomized study by Antonini et al. (2021), some dimensions of nurses’ quality of working life showed favorable changes following the intervention, particularly the alignment between everyday work activities and personal and professional values. However, these findings were not uniform across all quality-of-working-life dimensions.

Adverse events were rarely and inconsistently assessed. Serrada-Tejeda et al. (2026) prospectively monitored discomfort, dizziness, instability, falls, and physical injuries during the aging-simulation intervention and reported no adverse events or significant physical discomfort. Murray et al. (2015) also explicitly reported that no adverse events occurred. Antonini et al. (2021) acknowledged that the intervention could elicit strong emotional responses and made psychological support available to participants, but no intervention-related psychological harms were reported. In one simulation-based compassion program, noncompletion was partly attributed to workload and irregular attendance, suggesting a potential feasibility burden rather than a documented adverse effect (Hussien et al., 2025b).

The remaining studies did not systematically assess intervention-related distress, emotional burden, simulation-related discomfort, workload, or other unintended consequences. Therefore, the absence of reported harms should not be interpreted as evidence that these interventions are free of adverse or burdensome effects.

### 3.9. Risk of Bias

Risk-of-bias concerns varied according to study design, outcome source, and the degree to which intervention allocation could have influenced outcome measurement. Risk-of-bias judgments differed substantially by study design. Among the 19 individually randomized trials assessed using RoB 2, three (15.8%) were judged to be at low risk of bias, 13 (68.4%) raised some concerns, and three (15.8%) were judged to be at high risk of bias for the selected relational result. Of the two cluster-randomized trials assessed using the RoB 2 cluster-randomized trial extension, one raised some concerns and one was judged to be at high risk of bias. All 12 nonrandomized comparative studies assessed using ROBINS-I were judged to be at serious risk of bias. Detailed outcome-level judgments and supporting rationales are provided in Supplementary Tables S5–S7.

Among individually randomized trials, the randomization process was generally described, although allocation concealment and the timing of participant enrollment relative to randomization were not always reported in sufficient detail. Because most interventions were educational or behaviorally oriented, participants and intervention providers could not usually be blinded. This limitation was particularly relevant when the eligible outcome was based on professional self-report, self-rated confidence, or perceived competence.

Bias arising from deviations from the intended interventions was generally less concerning when analyses retained participants in their assigned groups. However, several reports provided insufficient information about protocol deviations, intervention adherence, or the analytical treatment of participants who did not complete training. Missing outcome data were particularly relevant in studies with longer follow-up or repeated patient assessments. In some studies, attrition, incomplete attendance, or loss of clinical encounters reduced the evaluable sample, and the potential influence of these losses on the estimated intervention effect could not be excluded.

Risk of bias in outcome measurement depended strongly on the assessment source. Professional self-reported empathy, compassion, caring competence, and humanistic-practice outcomes were susceptible to awareness of intervention allocation, expectancy effects, and socially desirable responding. Standardized-patient assessments and independently coded clinical encounters provided more direct evidence of behavioral performance, although assessor blinding and inter-rater reliability were not consistently documented. Patient- and family-reported outcomes were less directly affected by professional self-reporting, but some analyses involved patients clustered within clinicians or wards and did not always provide sufficient information about how this dependence was addressed.

Concerns regarding selection of the reported result arose when studies evaluated multiple instruments, subscales, behavioral categories, or assessment points without an accessible prespecified analysis plan. These concerns were more pronounced when only selected statistically significant findings were emphasized or when reporting differed across companion publications. Nevertheless, the companion reports by Fallowfield et al. (2002, 2003) and by Gibon et al. (2013) and Merckaert et al. (2015) provided complementary outcome or follow-up information and were treated as reports from the same underlying studies rather than as independent evidence.

Cluster-randomized trials presented additional concerns related to participant identification and recruitment after cluster allocation, baseline imbalances between clusters, the limited number of participating clusters, and the statistical handling of within-cluster correlation. These issues reduced confidence in estimates when clustering was not clearly incorporated into the analysis or when withdrawals occurred at the cluster level.

The nonrandomized comparative studies were particularly vulnerable to confounding and selection bias. Frequent limitations included pre-existing differences between intervention and comparator groups, self-selection into training, allocation based on workplace or organizational criteria, concurrent changes in clinical practice, and incomplete adjustment for professional or institutional characteristics. Consequently, observed between-group differences could not always be attributed exclusively to the educational intervention.

Overall, the evidence was most credible when allocation procedures were adequately reported, outcome assessors were independent or blinded, analyses accounted for clustering and baseline differences, and findings were based on observed behavior or patient-reported experience. Confidence was lower for uncontrolled organizational comparisons, professional self-reported outcomes, and analyses affected by attrition, inadequate adjustment, or selective reporting.

## 4. Discussion

This systematic review identified 33 independent comparative studies, reported in 35 publications, evaluating educational and professional development interventions intended to strengthen humanistic and relational practice among patient-facing healthcare professionals. The evidence was organized into three analytical domains: clinical empathy and emotional responsiveness; compassion, caring, and humanistic practice; and trust, rapport, and therapeutic relationships. Across these domains, favorable findings were more consistent for proximal professional outcomes and selected observable behaviors than for patient- or family-reported relational experience or sustained clinical change. However, the pattern and strength of the evidence differed across the three domains and should therefore be interpreted separately.

This pattern is broadly consistent with previous reviews showing that empathy, compassion, and communication training can produce favorable educational outcomes among healthcare students and professionals (Edwards et al., 2025; H. Ngo et al., 2025; H. L. Ngo et al., 2025; Peimani et al., 2025). However, the present findings qualify that evidence by showing that the apparent effect of training depends substantially on the assessment source and the proximity of the outcome to routine clinical care. Improvements in perceived ability or standardized educational performance should not be interpreted as equivalent to sustained behavioral change or improved patient experience.

### 4.1. Interpretation by Analytical Domain

This review extends previous systematic reviews in several respects. Earlier syntheses have generally focused on a single construct, professional group, educational approach, or clinical context, and many have included undergraduate students or uncontrolled pre–post studies (Edwards et al., 2025; Joo et al., 2025; McDarby et al., 2025; H. Ngo et al., 2025; H. L. Ngo et al., 2025; Peimani et al., 2025; Pira et al., 2025). In contrast, the present review was restricted to comparative interventions involving practicing, patient-facing healthcare professionals and integrated evidence across three related analytical domains. It also examined findings according to assessment source and distinguished proximal professional or educational outcomes from observable clinical behavior and patient- or family-reported experience. By additionally considering persistence over time and transfer to routine practice, the review identifies where favorable educational findings are supported by evidence of clinical application and where this progression remains uncertain.

#### 4.1.1. Clinical Empathy and Emotional Responsiveness

Clinical empathy and emotional responsiveness constituted the largest and most methodologically diverse domain. Favorable findings were observed for professional self-reported empathy, standardized educational performance, and specific behaviors such as emotional cue recognition, empathic responding, supportive communication, warmth, and involvement. These findings are broadly consistent with previous reviews reporting that empathy-focused education can improve cognitive and affective empathy, particularly when active, experiential, reflective, or patient-involved learning strategies are used (Edwards et al., 2025; H. Ngo et al., 2025; H. L. Ngo et al., 2025). However, the present review qualifies these findings by showing that improvements depended substantially on how empathy was assessed. Favorable effects were more consistent for proximal professional or educational outcomes than for empathy experienced by patients or demonstrated consistently during routine clinical encounters. Moreover, longer-term evidence suggested that some communication behaviors could be maintained while expressions of empathy declined over time. Empathy training should therefore not be evaluated exclusively through professional self-report or immediate post-training performance.

#### 4.1.2. Compassion, Caring, and Humanistic Practice

Evidence concerning compassion, caring, and humanistic practice was more limited and was concentrated primarily in nursing. Several interventions reported favorable changes in compassion competence, caring attitudes, self-efficacy, perceived patient-centered care, and humanistic-practice ability. Some studies also identified improvements in observed therapeutic communication or caring behaviors. These findings support the view that caring and compassionate practice may be influenced through simulation, storytelling, mentoring, reflective education, and theoretically grounded training. Nevertheless, most outcomes were assessed through professional or educational measures, and evidence derived directly from patients was scarce. Previous reviews have similarly found that educational programs more commonly assess perceived competence than collaboration, professional values, or care as experienced by patients and families (Bardaie et al., 2026; Joo et al., 2025). The concentration of this evidence within nursing also limits its direct transferability to other professional groups. The findings, therefore, support the potential educational value of these interventions but provide less certain evidence regarding changes in routine compassionate care.

#### 4.1.3. Trust, Rapport, and Therapeutic Relationships

The evidence concerning trust, rapport, and therapeutic relationships was the smallest of the three domains. Training produced favorable findings for selected trust-building, rapport-related, autonomy-supportive, and need-supportive skills. However, global patient trust and working alliance did not improve consistently. This distinction is important because trust and therapeutic alliance are broader relational outcomes that develop across repeated interactions and are influenced by professional behavior, patient expectations, continuity, clinical context, and organizational conditions. Consequently, improvement in an isolated communication behavior should not be interpreted as equivalent to improvement in the therapeutic relationship. Reviews of relational competence and professional–patient relationships have similarly emphasized that these outcomes emerge through interactions among individual, interpersonal, and organizational factors (Hansen et al., 2025; Neal et al., 2026; van Erp et al., 2025). Interventions targeting this domain may therefore require reinforcement, continuity, and supportive clinical environments in addition to individual skills training. Organizational compassion and supportive professional relationships may also contribute to environments in which trust-building and relational skills can be maintained, although organizational interventions remain insufficiently defined and evaluated (Nielsen et al., 2025).

### 4.2. Assessment Source and Interpretation of Intervention Effects

The source of outcome assessment was central to the interpretation of the evidence. Professional self-reports captured participants’ perceptions of their empathy, compassion, confidence, self-efficacy, or caring competence. These outcomes are relevant because they may reflect readiness to apply newly acquired knowledge and skills. Nevertheless, participants were generally aware of receiving the intervention, making these measures susceptible to expectation, social-desirability, and response-shift effects. Confidence-based measures should therefore not be treated as substitutes for performance-based assessments of competence (Jagemann et al., 2025).

Standardized-patient assessments and observer-coded clinical interactions provided more direct evidence of behavioral performance. Several studies reported improvements in emotional language, supportive utterances, warmth, involvement, open questioning, or responses to patient cues. However, performance during simulation or role-play does not necessarily demonstrate transfer to routine clinical care. Even assessments conducted in real consultations were heterogeneous in their coding systems, behavioral definitions, and denominators. This measurement variability has also been identified in the broader literature on nurse–patient communication and limits direct comparison across interventions (Al Sulaimi et al., 2026).

Patient- and family-reported outcomes most directly represented how relational care was experienced by its intended recipients. Nevertheless, these outcomes were assessed less frequently than professional or observer-based outcomes, and favorable between-group differences were less consistent across studies. Patient experience is influenced not only by individual professional behavior but also by continuity, organizational processes, previous expectations, clinical complexity, and the wider care environment (Dey et al., 2025; Quigley et al., 2024). Therefore, the three assessment sources should be considered complementary rather than hierarchically interchangeable. Future evaluations should combine professional self-assessment, independent behavioral observation, and patient- or family-reported experience to determine whether improvements converge across perspectives (Bardaie et al., 2026; Ta et al., 2026).

### 4.3. Transfer to Patient Experience and Relational Outcomes

Some included studies provided evidence that professional training may translate into outcomes perceived by patients. Favorable findings were reported for patient-rated empathy, psychosocial interaction, satisfaction, and selected patient-experience indicators in the studies by Riess et al. (2012), Smith et al. (1995), and Boissy et al. (2016). However, global trust and working alliance did not clearly improve in the trials by Thom (2000) and Meystre et al. (2013), despite changes in some targeted communication behaviors.

This distinction is important because complex relational outcomes may require more than the acquisition of isolated communication skills. Trust, alliance, and the experience of compassionate care develop across interactions and may depend on consistency, continuity, organizational support, and alignment between professional behavior and patient expectations. Previous reviews have similarly found that communication interventions more consistently improve proximal communication outcomes than downstream patient-care outcomes (McDarby et al., 2025). An updated meta-analysis of interventions targeting the patient–clinician relationship found a small favorable effect across health and healthcare outcomes, but the certainty of evidence was low, and heterogeneity was substantial (Neal et al., 2026). The present findings are compatible with that interpretation: training may contribute to improved relational care, but a consistent progression from professional learning to improved patient experience was not demonstrated.

### 4.4. Persistence and Clinical Transfer

The limited follow-up evidence distinguished immediate learning from sustained relational practice. Several interventions produced favorable outcomes immediately after training or during short-term evaluation, whereas maintenance at later assessments was inconsistent. In the Cancer Research UK trial, some trained communication behaviors remained evident at long-term follow-up, but expressions of empathy declined relative to the initial assessment (Fallowfield et al., 2002, 2003). Dong et al. (2026) similarly found that the immediate improvement in skills for addressing medical mistrust was not maintained at six months. Tai and Lin (2026) reported favorable ward-level patient outcomes at three months, but these differences were no longer evident at six months.

These findings suggest that a single educational exposure may not be sufficient to embed relational behaviors within routine practice. Reinforcement sessions, feedback based on observed interactions, supervised clinical application, peer reflection, and supportive organizational conditions may help maintain change. However, the reviewed studies did not provide sufficient comparative evidence to determine which reinforcement strategy is most effective. Limited follow-up has also been identified in reviews of arts-based, technology-supported, and communication-focused interventions (Mojarrad et al., 2026; Peimani et al., 2025; Pira et al., 2025). Future trials should therefore distinguish immediate acquisition, short-term clinical application, and longer-term maintenance through prespecified assessment points.

### 4.5. Methodological Quality and Implications for Future Research

Confidence in the evidence was limited by incomplete reporting of randomization and allocation procedures, outcomes susceptible to participants’ awareness of intervention status, attrition during longer follow-up, selective reporting across multiple subscales or assessment points, and inadequate handling of clustering in some studies. The nonrandomized evidence was additionally vulnerable to baseline differences, self-selection into training, concurrent institutional changes, and residual confounding. Consequently, large pre–post differences in self-reported outcomes should be interpreted more cautiously than adjusted between-group estimates derived from randomized studies.

The heterogeneity of interventions also prevented identification of a clearly superior educational approach. Simulation, role-play, narrative methods, workshops, mentoring, Balint groups, mindfulness, and technology-supported training have all produced favorable findings in some contexts (Edwards et al., 2025; Gong et al., 2024; H. Ngo et al., 2025; Pira et al., 2025; Xu et al., 2025). However, intervention intensity, comparator conditions, professional groups, outcome constructs, and assessment times differed substantially. Active comparators also meant that several trials evaluated the incremental value of one educational format over another rather than training versus no intervention. Null or modest between-group differences should therefore not automatically be interpreted as absence of learning.

Future studies should prespecify a limited set of clearly defined relational outcomes, distinguish proximal educational indicators from clinical and patient-related outcomes, and report adjusted between-group estimates with confidence intervals. Cluster-randomized studies should account for recruitment after allocation and professional-, ward-, or site-level clustering. Evaluations should include independent observation of routine clinical encounters, patient or family perspectives, and sufficiently long follow-up. Process evaluations would also help determine whether an intervention was delivered as intended and whether organizational conditions enabled professionals to apply the acquired skills.

### 4.6. Strengths and Limitations of the Review

This review has several methodological strengths. It integrated comparative evidence across three related but distinct domains of humanistic and relational practice and classified outcomes according to both their relational construct and assessment source. Professional self-report, standardized educational assessment, observer-rated behavior, and patient- or family-reported experience were examined separately. Study selection, data extraction, and risk-of-bias assessment were conducted independently by two reviewers, with arbitration by a third reviewer when required. Design-specific risk-of-bias tools were applied, companion publications were linked at the study level to avoid double counting, and intervention effects were distinguished from observational associations reported within the same studies.

Several limitations should also be acknowledged. The broad conceptual scope of the review resulted in substantial heterogeneity in relational constructs, measurement instruments, professional groups, intervention approaches, comparator conditions, assessment sources, and follow-up periods. This heterogeneity prevented a methodologically appropriate meta-analysis. The distribution of evidence was also uneven. Professional self-reported and standardized educational outcomes were more frequently represented than independently observed clinical behaviors or patient- and family-reported outcomes. Evidence from longer follow-up periods was limited, and some analytical domains were concentrated within particular professions or clinical settings.

The methodological quality of the included evidence imposed additional limitations. Most individually randomized trials raised some risk-of-bias concerns, one of the two cluster-randomized trials was judged to be at high risk of bias, and all 12 nonrandomized comparative studies were judged to be at serious risk of bias. Common concerns included incomplete reporting of allocation procedures, reliance on outcomes assessed by participants who were aware of their intervention status, attrition, insufficient adjustment for confounding, and selective reporting across multiple outcomes or assessment times.

The search covered four major biomedical and multidisciplinary databases but did not include the Cumulative Index to Nursing and Allied Health Literature (CINAHL). Relevant studies indexed primarily in this database may therefore have been missed. The restriction to English-language publications also introduced a potential risk of language bias. In addition, no formal certainty-of-evidence framework was applied because the evidence could not be organized into sufficiently homogeneous and clinically coherent intervention–comparison–outcome groups. The review therefore did not produce outcome-specific Grading of Recommendations Assessment, Development and Evaluation ratings. Finally, adverse and unintended effects were infrequently and inconsistently assessed, limiting the available evidence concerning intervention-related burden or harm.

## 5. Conclusions

This systematic review evaluated whether educational and professional development interventions can strengthen humanistic and relational practice among patient-facing healthcare professionals across three analytical domains: clinical empathy and emotional responsiveness; compassion, caring, and humanistic practice; and trust, rapport, and therapeutic relationships.

For clinical empathy and emotional responsiveness, favorable findings were most consistent for proximal professional outcomes and selected observer-rated communication behaviors, whereas patient- and family-reported effects were less frequently assessed and less consistently favorable. For compassion, caring, and humanistic practice, several interventions improved professional knowledge, attitudes, self-efficacy, compassion competence, caring behaviors, or humanistic-practice ability, although the evidence was concentrated largely in nursing and often relied on professional or educational assessments. For trust, rapport, and therapeutic relationships, proximal trust-building and rapport-related skills improved more consistently than global patient trust or working alliance, and sustained effects were uncommon.

Across all three domains, improvements in perceived competence, confidence, or self-reported relational ability should not be interpreted as equivalent to demonstrated changes in routine clinical behavior, patient or family experience, trust, or therapeutic alliance. Observer-based and patient-reported outcomes provided more direct evidence of clinical transfer, but these outcomes were less frequently available and findings were heterogeneous.

The available evidence did not identify a clearly superior intervention approach. Future evaluations should therefore combine professional self-assessment with independent behavioral observation and patient- or family-reported outcomes, incorporate longer follow-up periods, and use designs capable of establishing whether short-term gains in relational competence translate into sustained improvements in clinical practice. Overall, educational and professional development interventions appear promising for strengthening selected dimensions of humanistic and relational care, but conclusions regarding sustained clinical effectiveness should remain cautious until more robust and multidimensional evidence is available.

## Figures and Tables

**Table 1:** Characteristics of included studies.

Study	Primary Domain	Country	Study Design	Participants	Intervention/Comparator	Outcome Source and Timing	Main Comparative Finding
Beach et al. (2018)	Clinical empathy and emotional responsiveness	United States	Randomized comparative-intensity study.	12 HIV clinicians after a common one-day motivational interviewing workshop.	Workshop plus 3–5 personalized feedback rounds versus workshop alone.	Audio-coded patient-centeredness and affect; patient-rated MI consistency; pre/post with comparison of feedback intensity.	Across both arms, visits became more motivational interviewing (MI)-consistent (6.86 vs. 6.65, *p* = 0.005). Visits also became more patient-centered (1.34 vs. 0.96, *p* = 0.003) and showed more positive patient affect (22.36 vs. 20.84, *p* < 0.001). Intensive feedback provided no clear additional benefit.
Boissy et al. (2016)	Clinical empathy and emotional responsiveness	United States	Large nonrandomized controlled observational study	1537 trained attending physicians and 1951 untrained physicians.	Eight-hour relationship-centered communication course with didactics, demonstrations, and skills practice versus nonparticipation.	CGCAHPS/HCAHPS patient-experience scores; Jefferson Scale of Empathy; burnout and self-efficacy; immediate and ≥3-month professional outcomes.	Adjusted CGCAHPS communication scores were modestly higher after training (92.09 vs. 91.09, *p* < 0.03). HCAHPS Respect scores improved (91.08 vs. 88.79, *p* = 0.02). Self-reported empathy increased from 116.4 ± 12.7 to 124.0 ± 11.9 (*p* < 0.001).
Butow et al. (2008)	Clinical empathy and emotional responsiveness	Australia	RCT.	30 oncologists from six teaching hospitals.	1.5-day workshop plus four monthly 1.5 h videoconferences versus no CST.	Videotaped simulated-patient interviews coded for creating-environment, eliciting/responding to cues, and blocking behaviors; stress/burnout; post-training and 6 months.	Intervention physicians showed more environment-creating behaviors and fewer blocking responses at both follow-ups. Many between-group differences did not reach statistical significance.
Canivet et al. (2014)	Clinical empathy and emotional responsiveness	Belgium	RCT.	115 oncology nurses.	General communication-skills training with pain-management modules versus control.	Observed simulated cancer-pain interviews rated by two psychologists; content and relational behaviors after training/3 months.	Trained nurses asked more relevant questions and demonstrated better pain-focused assessment and communication behaviors. Improvements were not consistent across all relational behaviors.
Caris-Verhallen et al. (2000)	Clinical empathy and emotional responsiveness	Netherlands	Nonrandomized controlled study	40 nurses and 316 videotaped encounters in elder care.	Video Interaction Analysis training versus control.	Independent observer ratings of information provision, questioning, involvement, warmth, patronizing behavior, and support for self-care.	Training was associated with the provision of more nursing and health information and the use of more open-ended questions. Trained nurses demonstrated greater involvement and warmth and less patronizing behavior.
Du et al. (2025)	Clinical empathy and emotional responsiveness	China	Randomized controlled educational study.	46 licensed obstetric residents (23 per group).	Case-based narrative medicine education during an obstetric rotation versus conventional clinical teaching.	Narrative competency questionnaire, OSCE, knowledge testing, and teaching satisfaction after the rotation.	The intervention group obtained higher objective structured clinical examination (OSCE) and narrative-competency scores. Teaching satisfaction was also higher in the intervention group. All reported differences were statistically significant (*p* < 0.05).
Fallowfield et al. (2002, 2003)	Clinical empathy and emotional responsiveness	United Kingdom	Factorial RCT; companion reports from the same study	160 oncologists from 34 centers and 2407 videotaped patient consultations.	Three-day communication course and/or written feedback versus feedback alone/control.	Objective and subjective ratings of real consultations; primary outcomes were observed key communication behaviors. Companion report: Observed communication behaviors in real consultations, including empathy and patient-centered behaviors, at long-term follow-up.	The course improved several observed communication competencies during real consultations. Written feedback alone produced less consistent benefits. Several trained behaviors remained improved at longer-term or clinical-transfer assessment. Expressions of empathy declined at long-term follow-up (reported 54%, *p* = 0.001), indicating incomplete maintenance.
Fisher et al. (2014)	Clinical empathy and emotional responsiveness	United States	Nonrandomized controlled repeated-measures study.	Intervention received a one-hour simulation and controls viewed a one-hour video.	Four Habits emotion-focused nurse–parent communication session versus video control.	Self-reported preparation, communication skills, relationships, and confidence; reported clinical use of Four Habits.	Intervention nurses improved in four of the five assessed areas: preparation, communication, relationships, and confidence. More than half reported using at least one of the acquired habits in clinical practice.
Gibon et al. (2013); Merckaert et al. (2015)	Clinical empathy and emotional responsiveness	Belgium	Team-randomized controlled study; companion reports from the same study	80 radiotherapy team members.	Thirty-eight-hour communication-skills program versus waiting list.	Observed simulated-patient communication and self-efficacy at baseline and about 4 months. Companion report: Observed communication in real consultations and patient satisfaction at the end of radiotherapy.	Training increased assessment utterances (RR = 1.69), empathy (RR = 4.05), negotiation (RR = 2.34), emotional-word use (RR = 1.32), and team-resource content (RR = 1.38). Reported *p* values ranged from <0.001 to 0.037. At longer-term or clinical-transfer assessment, training increased assessment and supportive communication behaviors during workplace consultations. Patient-level effects were less consistent.
Haskard et al. (2008)	Clinical empathy and emotional responsiveness	United States	Controlled physician-training study with repeated patient assessments.	Sample size not reported in the extracted table.	Communication training designed to improve patient-centered consultation behavior versus control condition.	Patient satisfaction and perceptions of explanations/information; physician satisfaction/stress; global observer ratings.	Training improved patients’ perceptions of explanations and information provision. Overall patient-satisfaction patterns also favored training. Effects varied across assessment occasions.
Hussien et al. (2025a)	Clinical empathy and emotional responsiveness	Saudi Arabia/Egypt collaboration	Controlled quasi-experimental study.	124 staff nurses (62 per group).	Standardized-patient simulation-based compassion training versus control/usual training.	Emotional intelligence, empathy, and perceived quality of patient care; professional self-report before and after training.	The intervention group showed marked post-training improvements in emotional intelligence and empathy. Perceived quality of care was also more favorable after training. The reported between-group and post-training contrasts were statistically significant.
Kruijver et al. (2001)	Clinical empathy and emotional responsiveness	Netherlands	Nonrandomized controlled study	46 ward nurses completed 92 simulated admission interviews with patients with cancer.	Communication program focused on discussing and handling emotions versus control.	Videotaped instrumental and affective communication in simulated interviews.	Trained nurses demonstrated greater affective communication than controls. Emotion-handling behaviors also improved in the intervention group.
Liang et al. (2025)	Clinical empathy and emotional responsiveness	China	RCT	117 third-year oncology residents (SST-AIDET n = 54; traditional teaching n = 63).	Situational simulation teaching integrating AIDET, theoretical teaching, simulation, and discussion versus teacher-centered instruction.	SEGUE, CPPC-AS, and CARE during an OSCE; standardized-patient/assessor-based post-training evaluation.	The intervention group had a higher median SEGUE score after training (22 vs. 18). Median CARE scores were also higher (45 vs. 43). CPPC-AS task, performance, and total scores favored the intervention group. All reported differences were statistically significant (*p* < 0.001).
Liénard et al. (2010)	Clinical empathy and emotional responsiveness	Belgium	Wait-list-controlled RCT	88 residents.	Forty-hour communication-skills program versus waiting list.	Patient satisfaction after visits and observed communication in audiotaped real inpatient rounds.	Patients treated by trained residents reported greater communication satisfaction (median 92 vs. 88). Trained residents used more assessment and supportive utterances during clinical rounds.
Nixon et al. (2020)	Clinical empathy and emotional responsiveness	Australia	Partially randomized three-arm controlled study.	147 cancer-care professionals from medicine, nursing, and allied health (control 50; CARE Express 48; CARE 49).	Two-hour CARE Express or one-day CARE workshop versus no training.	Confidence in identifying/responding to emotions; written responses to a difficult encounter; Physician Belief Scale; baseline, post-training, and 3 months.	Both active intervention groups improved in confidence, psychosocial orientation, and acknowledgment responses through 3 months. No material differences were observed between the two active intervention formats.
Pollak et al. (2016)	Clinical empathy and emotional responsiveness	United States	RCT	46 pediatric/family physicians and 527 encounters with adolescents with overweight.	Individually tailored online motivational-interviewing intervention, followed by a report phase, versus comparison arm.	Observer-coded empathy/MI spirit and behaviors; patient-rated empathy and motivation.	Intervention-phase comparisons favored training for observer-rated empathy and MI spirit (both *p* < 0.001). Training also favored open-ended questions (*p* = 0.02) and MI-consistent behaviors (*p* = 0.04). Greater observed empathy was associated with greater patient motivation.
Rask et al. (2009)	Clinical empathy and emotional responsiveness	Denmark	Controlled oncology outpatient nursing study.	Sample size not reported in the extracted table.	Training aimed at improving nurse–patient communication versus comparison care.	Observed communication behaviors and nurse self-efficacy; patient-related outcomes reported in the full text.	The hypothesized improvements in nurses’ communication and self-efficacy were not consistently supported. Effects were limited across the measured outcomes.
Razavi et al. (2002)	Clinical empathy and emotional responsiveness	Belgium	RCT	Clinical and simulated interviews assessed before, immediately after, and 3 months after training.	Communication workshop emphasizing emotional language and response versus control.	Frequency and repertoire of emotional words used by nurses and elicited from patients; observer/content-analysis outcomes.	Emotional-word use increased most clearly during simulated encounters. Training facilitated patients’ emotional expression at 3 months (reported *p* = 0.005). Effects during real clinical interviews were weaker.
Riess et al. (2012)	Clinical empathy and emotional responsiveness	United States	RCT	Sample size not reported in the extracted table.	Brief neuroscience-informed empathy curriculum versus control.	Patient-rated CARE; empathy-neuroscience knowledge; facial-emotion recognition.	Change in patient-rated CARE scores favored training by 2.2 points (*p* = 0.04). Empathy-related knowledge improved by 1.8 points (*p* < 0.001). Facial-emotion recognition improved by 1.9 points (*p* < 0.001).
Serrada-Tejeda et al. (2026)	Clinical empathy and emotional responsiveness	Spain	RCT	82 healthcare professionals from four nursing homes (41 per group).	Common education on empathy/aging in both groups; experimental group additionally used the GERT aging-simulation suit.	Self-reported Interpersonal Reactivity Index and clinical-empathy-related outcomes after training.	No between-group difference was found in Interpersonal Reactivity Index empathy scores. The simulation group improved in selected secondary perception and experience measures.
Antonini et al. (2021)	Compassion, caring, and humanistic practice	Switzerland	cluster RCT.	101 hemodialysis nurses in 10 hospitals.	Brief Watson Human Caring-based educational intervention versus control.	Caring Nurse–Patient Interaction Scale (EIIP-70) and quality of working life at four time points.	The intervention reinforced selected humanistic caring attitudes and behaviors. Effects varied across EIIP-70 dimensions and assessment times. Work-related quality-of-life outcomes were secondary.
Hussien et al. (2025b)	Compassion, caring, and humanistic practice	Saudi Arabia	controlled quasi-experimental study.	100 nurses.	Simulation-based compassionate-care training versus control.	Caregiving behavior, self-efficacy, and compassion competence (communication, sensitivity, insight) before/after training.	Mean caregiving-behavior scores increased from 44.20 to 77.32 in the intervention group. Mean self-efficacy scores increased from 21.16 to 33.72. Significant improvements were reported across compassion domains. The control group showed minimal change.
Mau et al. (2025)	Compassion, caring, and humanistic practice	Indonesia	Controlled quasi-experimental pretest–posttest study	120 inpatient nurses (60 per group).	Structured training plus mentoring over 3 months versus control.	Validated questionnaires assessing caring knowledge, attitudes, and actions.	Large improvements favored intervention for knowledge (partial η^2^ = 0.882) Attitudes (0.711), and actions (0.737) Overall between-group improvement was reported (*p* < 0.001).
Park et al. (2021)	Compassion, caring, and humanistic practice	South Korea	Controlled quasi-experimental study	88 nurses (experimental 39; control 49).	Patient-centered-care program using patient-role enactment and storytelling versus delayed training.	Perceived PCC, compassion, and knowledge transfer before/after training.	Improvement in perceived patient-centered care was greater in the intervention group (*p* = 0.001). Improvement in compassion also favored the intervention group (*p* = 0.006). Knowledge transfer correlated with patient-centered care (r = 0.55) and compassion (r = 0.63).
Shimran and Ali (2025)	Compassion, caring, and humanistic practice	Iraq	Controlled quasi-experimental study	110 critical-care nurses (55 per group).	Integrated ethics and therapeutic-communication educational program versus no program.	Validated 72-item knowledge questionnaire and 34-item observed/practice tool; pre/post assessment.	Knowledge improved in the intervention group relative to the control group. Observed practice also improved relative to the control group. The reported differences were statistically significant (*p* < 0.05).
Tai and Lin (2026)	Compassion, caring, and humanistic practice	Taiwan	Ward-clustered quasi-experimental pretest–posttest study.	Eight wards; 60 intervention and 61 control nurses.	Structured experiential psychological-support communication training versus no training.	Nurse knowledge, attitudes, skills, confidence, and job stress at baseline, 3, and 6 months; ward-aggregate patient satisfaction and quality of life.	Most assessed nursing competencies showed favorable group-by-time interactions. Ward-level patient satisfaction and quality of life favored the intervention wards at 3 months. These ward-level differences were not maintained at 6 months.
Zhuang et al. (2025)	Compassion, caring, and humanistic practice	China	RCT	77 newly recruited ophthalmic nurses (39 intervention; 38 control).	DIKW-based visual-impairment simulation versus traditional training.	Nurses Humanistic Practice Ability Scale and Professional Identity Scale at baseline and post-intervention.	Post-intervention humanistic-practice ability favored the training group (t = 3.132, *p* < 0.05). Professional identity also favored the training group (Z = −5.222, *p* < 0.001).
Dong et al. (2026)	Trust rapport, and therapeutic relationship	United States	pilot RCT	59 HIV-care providers (29 training; 30 control).	Online training using validation and motivational interviewing to address intersectional stigma and medical mistrust versus no training.	Survey and role-play skills; training-consistent communication; post-training and 6 months.	Skills for addressing medical mistrust showed a moderate immediate improvement (d = 0.54). This improvement was not maintained at 6 months (d = −0.14). Role-play strategies showed a small improvement (d = 0.35). The largest differences were found for autonomy support (d = 1.26) and checking understanding (d = 0.66).
Meystre et al. (2013)	Trust rapport, and therapeutic relationship	Switzerland	Controlled intervention study	56 trained and 57 untrained oncology clinicians assessed in simulated interviews 6 months apart.	Communication-skills training versus no training.	Working Alliance Inventory–Short Revised and RIAS-coded communication.	The intervention did not improve working alliance. Positive talk and psychosocial counseling were associated with a stronger working alliance. Negative talk, biomedical information provision, and patient questions were associated with a weaker working alliance. These associations were observational and were not interpreted as intervention effects.
Murray et al. (2015)	Trust rapport, and therapeutic relationship	United Kingdom/Ireland	Cluster RCT.	24 physiotherapists and 24 patients with chronic low back pain.	Eight-hour self-determination-theory communication training versus wait-list control.	Blinded independent rating with the Health Care Climate Questionnaire of need-supportive behavior in an audiotaped clinical session.	Independent ratings strongly favored training (Cohen d = 2.27, *p* < 0.01).
Norfolk et al. (2009)	Trust rapport, and therapeutic relationship	United Kingdom	Pilot quasi-experimental study	37 general-practice trainees.	Short rapport-development training based on an empathic-skills model versus comparison group.	Rapport knowledge, attitudes, confidence, and behavior dimensions.	Training improved rapport-related knowledge. All three affective dimensions also improved. Transfer to observable behavior was more limited and variable.
Smith et al. (1995)	Trust rapport, and therapeutic relationship	United States	Randomized controlled study	Sample size not reported in the extracted table.	Intensive psychosocial-medicine training versus standard residency education.	Patient satisfaction, perceived empathy/trust-related aspects, and comparison with other physicians.	Patients treated by trained residents reported greater satisfaction than patients treated by control-group residents. Ratings of psychosocial interactions also favored trained residents. Effects varied across patient-rated dimensions
Thom (2000)	Trust rapport, and therapeutic relationship	United States	physician-randomized controlled study.	20 physicians (10 per group) with patient surveys before and 6 months after training.	Short workshop targeting seven trust-building behavior categories versus control.	Patient-reported physician behaviors, trust, satisfaction, and utilization.	Intervention physicians improved in 16 of the 19 targeted trust-building behaviors. The study did not demonstrate a statistically significant improvement in global patient trust.

Note: The table summarizes 33 independent studies reported across 35 publications. Fallowfield et al. (2002, 2003) and Gibon et al. (2013) and Merckaert et al. (2015) were treated as companion reports from the same underlying studies. Comparative findings and effect estimates are presented as reported by the original authors; adjusted between-group estimates were prioritized when available. Within-group changes are reported only when an appropriate comparative estimate was unavailable. AIDET, Acknowledge, Introduce, Duration, Explanation, and Thank You; CARE, Consultation and Relational Empathy; CGCAHPS, Clinician and Group Consumer Assessment of Healthcare Providers and Systems; CPPC-AS, Chinese Physician–Patient Communication Assessment Scale; DIKW, Data–Information–Knowledge–Wisdom; EIIP-70, 70-item Caring Nurse–Patient Interaction Scale; HCAHPS, Hospital Consumer Assessment of Healthcare Providers and Systems; MI, motivational interviewing; OSCE, objective structured clinical examination; PCC, patient-centered care; RCT, randomized controlled trial; RIAS, Roter Interaction Analysis System; RR, rate ratio; SEGUE, Set the Stage, Elicit Information, Give Information, Understand the Patient’s Perspective, and End the Encounter.

**Table 2:** Distribution and overall pattern of findings by primary relational domain.

Primary Relational Domain	Reports, n	Main Assessment Sources	Overall Pattern of Findings
Clinical empathy and emotional responsiveness	22 (20)	Cognitive and affective empathy; emotional cue recognition and response; empathic communication; emotional language; warmth and involvement.	Consultation and Relational Empathy measure; Jefferson Scale of Empathy; Interpersonal Reactivity Index; SEGUE framework; Chinese Physician–Patient Communication Assessment Scale; Roter Interaction Analysis System; study-specific coding of emotional cues and communication behaviors.
Compassion, caring, and humanistic practice	7	Compassion competence; caring attitudes and behaviors; humanistic-practice ability; patient-centered caring; therapeutic communication.	70-item Caring Nurse–Patient Interaction Scale; Nurses Humanistic Practice Ability Scale; compassion-competence and caregiving-behavior scales; study-specific knowledge, attitude, self-efficacy, and observed-practice measures
Trust, rapport, and therapeutic relationships	6	Trust-building; rapport; therapeutic or working alliance; autonomy support; need-supportive behavior; broader professional–patient relationships.	Working Alliance Inventory–Short Revised; Health Care Climate Questionnaire; patient trust and satisfaction measures; Roter Interaction Analysis System; role-play and observer-rated communication measures.

Note. Each report was assigned to one primary analytical domain for classification and counting. Reports assessing outcomes relevant to more than one domain could contribute to the corresponding narrative syntheses but were counted only once in this table. The listed instruments and assessment approaches are illustrative of the measures used within each domain. The Consultation and Relational Empathy measure and variants of the Jefferson Scale of Empathy were each used in two independent studies; most other named instruments were used in a single study. Instruments addressing similar constructs were not considered interchangeable when they differed in assessment source, operational definition, or measurement approach.

## Data Availability

No new primary data were generated in this systematic review. All data analyzed were obtained from the studies cited in the article. The search strategies and the study-level data supporting the findings are provided in the article and its Supplementary Materials. Additional review materials are available from the corresponding author upon reasonable request.

## Supplementary Materials

The following supporting information can be downloaded at https://www.mdpi.com/article/10.3390/ejihpe16090142/s1, Table S1. Complete search strategies used in each electronic database. Table S2. Study-level evidence on clinical empathy and emotional responsiveness. Table S3. Study-level evidence on compassion, caring, and humanistic practice. Table S4. Study-level evidence on trust, rapport, and therapeutic relationships. Table S5. Outcome-level RoB 2 assessments for individually randomized trials. Table S6. Outcome-level RoB 2 assessments for cluster-randomized trials. Table S7. Outcome-level ROBINS-I assessments for nonrandomized comparative intervention studies.
