# Supplementary material for: Educational and Professional Development Interventions to Strengthen Empathy, Compassion, and Relational Care Among Healthcare Professionals: A Systematic Review
Source: Eur J Investig Health Psychol Educ. 2026 Sep 17;16(9):142. doi: 10.3390/ejihpe16090142 (PMC13605427; doi:10.3390/ejihpe16090142)
Supplement: Supplementary file 1 [file ejihpe-16-00142-s001.zip › ejihpe-4502299-supplementary.pdf]

Table S1. Complete search strategies used in each electronic database

Database/platform	Complete search strategy	Records retrieved
**PubMed/MEDLINE**	(("healthcare professional*"[Title/Abstract] OR "health care professional*"[Title/Abstract] OR "healthcare provider*"[Title/Abstract] OR "health care provider*"[Title/Abstract] OR clinician*[Title/Abstract] OR nurse*[Title/Abstract] OR physician*[Title/Abstract] OR pharmacist*[Title/Abstract] OR "allied health professional*"[Title/Abstract] OR resident*[Title/Abstract]) AND ("training program*"[Title/Abstract] OR "educational program*"[Title/Abstract] OR "educational intervention*"[Title/Abstract] OR "training intervention*"[Title/Abstract] OR "professional development program*"[Title/Abstract] OR "communication training"[Title/Abstract] OR "communication skills training"[Title/Abstract] OR "empathy training"[Title/Abstract] OR "compassion training"[Title/Abstract] OR "humanization training"[Title/Abstract] OR "humanisation training"[Title/Abstract] OR "relational skills training"[Title/Abstract] OR "interpersonal skills training"[Title/Abstract] OR "simulation-based training"[Title/Abstract] OR "workshop-based training"[Title/Abstract]) AND ("humanization of care"[Title/Abstract] OR "humanisation of care"[Title/Abstract] OR "humanized care"[Title/Abstract] OR "humanised care"[Title/Abstract] OR "humanistic care"[Title/Abstract] OR "humanistic practice"[Title/Abstract] OR "person-centered care"[Title/Abstract] OR "person-centred care"[Title/Abstract] OR "patient-centered care"[Title/Abstract] OR "patient-centred care"[Title/Abstract] OR "relationship-centered care"[Title/Abstract] OR "relationship-centred care"[Title/Abstract] OR "relational care"[Title/Abstract] OR "compassionate care"[Title/Abstract] OR "empathic care"[Title/Abstract] OR "empathetic care"[Title/Abstract] OR "therapeutic relationship*"[Title/Abstract] OR "professional-patient relation*"[Title/Abstract] OR "provider-patient relation*"[Title/Abstract] OR "clinician-patient relation*"[Title/Abstract] OR "nurse-patient relation*"[Title/Abstract] OR "physician-patient relation*"[Title/Abstract])) AND humans[Filter] AND english[Language] AND medline[sb]	720
**Cochrane Central Register of Controlled Trials (CENTRAL)**	("healthcare professional*" OR "health care professional*" OR "healthcare provider*" OR "health care provider*" OR clinician* OR nurse* OR physician* OR pharmacist* OR "allied health professional*" OR resident*):ti,ab,kw AND ("training program*" OR "educational program*" OR "educational intervention*" OR "training intervention*" OR "professional development program*" OR "communication training" OR "communication skills training" OR "empathy training" OR "compassion training" OR "humanization training" OR "humanisation training" OR "relational skills training" OR "interpersonal skills training" OR "simulation-based training" OR "workshop-based training"):ti,ab,kw AND ("humanization of care" OR "humanisation of care" OR "humanized care" OR "humanised care" OR "humanistic care" OR "humanistic practice" OR "person-centered care" OR "person-centred care" OR "patient-centered care" OR "patient-centred care" OR "relationship-centered care" OR "relationship-centred care" OR "relational care" OR "compassionate care" OR "empathic care" OR "empathetic care" OR "therapeutic relationship*" OR "professional-patient relation*" OR "provider-patient relation*" OR "clinician-patient relation*" OR "nurse-patient relation*" OR "physician-patient relation*"):ti,ab,kw; Cochrane Library collection restricted to Trials.	653

**Web of Science Core Collection**	TS=(("healthcare professional*" OR "health care professional*" OR "healthcare provider*" OR "health care provider*" OR clinician* OR nurse* OR physician* OR pharmacist* OR "allied health professional*" OR resident*) AND ("training program*" OR "educational program*" OR "educational intervention*" OR "training intervention*" OR "professional development program*" OR "communication training" OR "communication skills training" OR "empathy training" OR "compassion training" OR "humanization training" OR "humanisation training" OR "relational skills training" OR "interpersonal skills training" OR "simulation-based training" OR "workshop-based training") AND ("humanization of care" OR "humanisation of care" OR "humanized care" OR "humanised care" OR "humanistic care" OR "humanistic practice" OR "person-centered care" OR "person-centred care" OR "patient-centered care" OR "patient-centred care" OR "relationship-centered care" OR "relationship-centred care" OR "relational care" OR "compassionate care" OR "empathic care" OR "empathetic care" OR "therapeutic relationship*" OR "professional-patient relation*" OR "provider-patient relation*" OR "clinician-patient relation*" OR "nurse-patient relation*" OR "physician-patient relation*")); refined by document type: Article and language: English.	266
**Scopus**	TITLE-ABS-KEY(("healthcare professional*" OR "health care professional*" OR "healthcare provider*" OR "health care provider*" OR clinician* OR nurse* OR physician* OR pharmacist* OR "allied health professional*" OR resident*) AND ("training program*" OR "educational program*" OR "educational intervention*" OR "training intervention*" OR "professional development program*" OR "communication training" OR "communication skills training" OR "empathy training" OR "compassion training" OR "humanization training" OR "humanisation training" OR "relational skills training" OR "interpersonal skills training" OR "simulation-based training" OR "workshop-based training") AND ("humanization of care" OR "humanisation of care" OR "humanized care" OR "humanised care" OR "humanistic care" OR "humanistic practice" OR "person-centered care" OR "person-centred care" OR "patient-centered care" OR "patient-centred care" OR "relationship-centered care" OR "relationship-centred care" OR "relational care" OR "compassionate care" OR "empathic care" OR "empathetic care" OR "therapeutic relationship*" OR "professional-patient relation*" OR "provider-patient relation*" OR "clinician-patient relation*" OR "nurse-patient relation*" OR "physician-patient relation*")) AND (LIMIT-TO(DOCTYPE,"ar")) AND (LIMIT-TO(LANGUAGE,"English"))	982

Table S2. Study-level evidence on clinical empathy and emotional responsiveness

Study	Setting, design, and sample	Intervention and comparator	Outcome, source, and timing	Findings reported in the PDF	Quantitative-synthesis implications
Liang et al. (2025)	China; randomized controlled educational study; 117 third-year oncology residents (SST-AIDET n=54; traditional teaching n=63).	Situational simulation teaching integrating AIDET, theoretical teaching, simulation, and discussion versus teacher-centered instruction.	SEGUE, CPPC-AS, and CARE during an OSCE; standardized-patient/assessor-based post-training evaluation.	Higher post-training median SEGUE (22 vs 18), CARE (45 vs 43), and CPPC-AS task, performance, and total scores in the intervention group (all reported p<.001).	CARE may be extractable if dispersion data are available; otherwise, narrative synthesis. Do not pool SEGUE/CPPC-AS with CARE.
Nixon et al. (2020)	Australia; partial randomization, three-arm controlled study; 147 cancer-care professionals from medicine, nursing, and allied health (control 50; CARE Express 48; CARE 49).	Two-hour CARE Express or one-day CARE workshop versus no training.	Confidence in identifying/responding to emotions; written responses to a difficult encounter; Physician Belief Scale; baseline, post-training, and 3 months.	Both active arms improved confidence, psychosocial orientation, and acknowledgement responses through 3 months; no material difference between the two active formats.	Written response and self-report outcomes should be synthesized separately. Multi-arm adjustment is required if both intervention arms enter one meta-analysis.
Hussien et al. (2025a)	Saudi Arabia/Egypt collaboration; controlled quasi-experimental study; 124 staff nurses (62 per group).	Standardized-patient simulation-based compassion training versus control/usual training.	Emotional intelligence, empathy, and perceived quality of patient care; professional self-report before and after training.	The intervention group showed marked post-training gains in emotional intelligence and empathy and more favorable perceived care quality; the PDF reports statistically significant between-group/post-training contrasts.	Potential SMD for empathy if group means and SDs are completely reported; retain perceived care quality as a distinct secondary outcome.
Boissy et al. (2016)	United States; large observational controlled evaluation at an academic medical	Eight-hour relationship-centered communication course with didactics,	CGCAHPS/HCAHPS patient-experience scores; Jefferson Scale of Empathy; burnout and	Adjusted CGCAHPS communication was modestly higher after training (92.09 vs 91.09, p<.03); HCAHPS	Because allocation was nonrandom and empathy was reported as within-participant

Study	Setting, design, and sample	Intervention and comparator	Outcome, source, and timing	Findings reported in the PDF	Quantitative-synthesis implications
	center; 1,537 trained attending physicians and 1,951 untrained physicians.	demonstrations, and skills practice versus nonparticipation.	self-efficacy; immediate and ≥3-month professional outcomes.	Respect improved (91.08 vs 88.79, p=.02). Self-reported empathy increased from 116.4±12.7 to 124.0±11.9 (p<.001).	change, do not combine uncritically with randomized post-test effects; ROBINS-I and adjusted-effect extraction required.
Du et al. (2025)	China; randomized educational study; 46 licensed obstetric residents (23 per group).	Case-based narrative medicine education during an obstetric rotation versus conventional clinical teaching.	Narrative competency questionnaire, OSCE, knowledge testing, and teaching satisfaction after the rotation.	The intervention group had higher OSCE and narrative-competency scores and greater teaching satisfaction (all reported p<.05).	Narrative competence is a humanistic proxy, not a direct empathy measure. Pool only with the same validated construct; otherwise narrative synthesis.
Fallowfield et al. (2003)	United Kingdom; 12–15-month companion follow-up of the Cancer Research UK oncologist trial.	Intensive communication-skills course, with/without written feedback, compared with feedback alone/control.	Observed communication behaviors in real consultations, including empathy and patient-centered behaviors, at long-term follow-up.	Several trained behaviors remained improved, but expressions of empathy declined at long-term follow-up (reported 54%, p=.001), indicating incomplete maintenance.	Companion report to study E11. Use for long-term follow-up only; never count as an independent sample alongside Fallowfield et al. (2002).
Riess et al. (2012)	United States; randomized controlled trial of resident physicians.	Brief neuroscience-informed empathy curriculum versus control.	Patient-rated CARE; empathy-neuroscience knowledge; facial-emotion recognition.	Change in patient-rated CARE favored training by 2.2 points (p=.04); knowledge and facial-emotion decoding also improved (differences 1.8 and 1.9; both p<.001).	CARE is potentially poolable with other patient-rated CARE outcomes if compatible follow-up and variance data are available.
Fisher et al. (2014)	United States; experimental repeated-measures study of newly licensed nurses; intervention received a	Four Habits emotion-focused nurse–parent communication session versus video control.	Self-reported preparation, communication skills, relationships, and confidence; reported	Intervention nurses improved in four of five assessed areas—preparation, communication, relationships, and	Primarily self-reported and proximal outcomes; narrative synthesis unless complete

Study	Setting, design, and sample	Intervention and comparator	Outcome, source, and timing	Findings reported in the PDF	Quantitative-synthesis implications
	one-hour simulation and controls viewed a one-hour video.		clinical use of Four Habits.	confidence—and more than half reported clinical use of at least one habit.	comparable scale data are available.
Merckaert et al. (2015)	Belgium; cluster/team-randomized study; four radiotherapy teams; 237 real planning sessions recorded.	Thirty-eight-hour team communication-skills program versus waiting list.	Observed communication in real consultations and patient satisfaction at the end of radiotherapy.	Training increased assessment and supportive communication behaviors in workplace consultations; patient-level effects were less consistent.	Companion report to study E08. Use real-clinical-transfer outcomes; account for team clustering and do not duplicate Gibon et al. (2013).
Liénard et al. (2010)	Belgium; randomized waiting-list controlled trial; 88 residents.	Forty-hour communication-skills program versus waiting list.	Patient satisfaction after visits and observed communication in audiotaped real inpatient rounds.	Patients of trained residents reported higher communication satisfaction (median 92 vs 88); trained residents also used more assessment and supportive utterances in clinical rounds.	Median-based outcome may require transformation or narrative synthesis. Observed behaviors are count/rate outcomes and should remain separate.
Razavi et al. (2002)	Belgium; randomized controlled study of oncology nurses; clinical and simulated interviews assessed before, immediately after, and 3 months after training.	Communication workshop emphasizing emotional language and response versus control.	Frequency and repertoire of emotional words used by nurses and elicited from patients; observer/content-analysis outcomes.	Emotional-word use increased most clearly in simulations and facilitated patient emotional expression at 3 months (reported p=.005); effects in real interviews were weaker.	Behavior-count outcome; use rate ratio only if denominators and variance are recoverable. Check whether this report belongs to a larger study family before pooling.
Gibon et al. (2013)	Belgium; team-randomized controlled study; 80 radiotherapy team members.	Thirty-eight-hour communication-skills program versus waiting list.	Observed simulated-patient communication and self-efficacy at	Training increased assessment utterances (RR 1.69), empathy (RR 4.05), negotiation (RR 2.34),	Companion report to study E08. Count/rate outcomes may be meta-analyzable only with

Study	Setting, design, and sample	Intervention and comparator	Outcome, source, and timing	Findings reported in the PDF	Quantitative-synthesis implications
			baseline and about 4 months.	emotional words (RR 1.32), and team-resource content (RR 1.38); reported p values ranged from <.001 to .037.	compatible coding systems; adjust for team randomization.
Haskard et al. (2008)	United States; controlled physician-training study with repeated patient assessments.	Communication training designed to improve patient-centered consultation behavior versus control condition.	Patient satisfaction and perceptions of explanations/information; physician satisfaction/stress; global observer ratings.	Training improved patient perceptions of explanations and information giving and overall satisfaction patterns, with effects varying over assessment occasions.	Extract adjusted repeated-measures estimates from the PDF tables. The relational construct is broader than empathy; likely narrative or patient-experience subgroup.
Canivet et al. (2014)	Belgium; randomized controlled trial; 115 oncology nurses.	General communication-skills training with pain-management modules versus control.	Observed simulated cancer-pain interviews rated by two psychologists; content and relational behaviors after training/3 months.	Trained nurses asked more relevant questions and displayed improved pain-focused assessment and communication behaviors; not all relational behaviors changed consistently.	Observer-coded count outcomes; pool only with studies using compatible coding and follow-up.
Kruijver et al. (2001)	Netherlands; controlled evaluation; 46 ward nurses completing 92 simulated admission interviews with patients with cancer.	Communication program focused on discussing and handling emotions versus control.	Videotaped instrumental and affective communication in simulated interviews.	Trained nurses increased affective communication and emotion-handling behaviors compared with controls.	Observer-coded behavioral outcomes; extract counts/rates and clustering by nurse. Do not combine with professional self-report.
Caris-Verhallen et al. (2000)	Netherlands; experimental/control evaluation; 40 nurses and 316 videotaped encounters in elder care.	Video Interaction Analysis training versus control.	Independent observer ratings of information provision, questioning, involvement, warmth, patronizing behavior,	Training was associated with more nursing/health information, more open questions, greater involvement and warmth,	Nonrandomized design limitations are acknowledged by the authors. ROBINS-I; adjusted multilevel estimates preferred.

Study	Setting, design, and sample	Intervention and comparator	Outcome, source, and timing	Findings reported in the PDF	Quantitative-synthesis implications
			and support for self-care.	and less patronizing behavior.	
Rask et al. (2009)	Denmark; controlled oncology outpatient nursing intervention.	Training aimed at improving nurse–patient communication versus comparison care.	Observed communication behaviors and nurse self-efficacy; patient-related outcomes reported in the full text.	The hypotheses concerning clear improvement in nurse communication and self-efficacy were not consistently supported; effects were limited across measured outcomes.	Retain null findings. Extract exact adjusted contrasts from tables; likely narrative synthesis because outcomes and reporting differ.
Butow et al. (2008)	Australia; randomized controlled trial; 30 oncologists from six teaching hospitals.	1.5-day workshop plus four monthly 1.5-hour videoconferences versus no CST.	Videotaped simulated-patient interviews coded for creating-environment, eliciting/responding to cues, and blocking behaviors; stress/burnout; post-training and 6 months.	Intervention doctors showed more environment-creating and fewer blocking behaviors at both follow-ups, but many between-group differences did not reach statistical significance.	Observed behaviors should be retained regardless of significance; count/rate extraction may support synthesis with compatible cue-response studies.
Beach et al. (2018)	United States; randomized comparative-intensity trial; 12 HIV clinicians after a common one-day motivational interviewing workshop.	Workshop plus 3–5 personalized feedback rounds versus workshop alone.	Audio-coded patient-centeredness and affect; patient-rated MI consistency; pre/post with comparison of feedback intensity.	Across both arms, visits became more MI-consistent (6.86 vs 6.65, p=.005), more patient-centered (1.34 vs 0.96, p=.003), and showed more positive patient affect (22.36 vs 20.84, p<.001); no clear added benefit of intensive feedback.	Because both groups received active training, this estimates incremental feedback rather than training versus no training. Use comparative arm effect, not pooled pre/post change.
Pollak et al. (2016)	United States; randomized trial; 46 pediatric/family physicians and 527	Individually tailored online motivational-interviewing intervention, followed	Observer-coded empathy/MI spirit and behaviors; patient-rated	Intervention-phase differences favored training for empathy and MI spirit (both p<.001), open questions	Clustered encounters within physicians. Association between empathy and

Study	Setting, design, and sample	Intervention and comparator	Outcome, source, and timing	Findings reported in the PDF	Quantitative-synthesis implications
	encounters with adolescents with overweight.	by a report phase, versus comparison arm.	empathy and motivation.	(p=.02), and MI-consistent behaviors (p=.04); higher observed empathy was associated with patient motivation.	motivation is not an intervention effect and should not be pooled as such.
Fallowfield et al. (2002)	United Kingdom; factorial randomized controlled trial; 160 oncologists from 34 centers and 2,407 videotaped patient consultations.	Three-day communication course and/or written feedback versus feedback alone/control.	Objective and subjective ratings of real consultations; primary outcomes were observed key communication behaviors.	The course improved several observed communication competencies in real consultations; written feedback alone produced less consistent benefit.	Primary report for study E11. Use short-term effect here and long-term outcomes from Fallowfield et al. (2003) without double-counting.
Serrada-Tejeda et al. (2026)	Spain; randomized controlled trial; 82 healthcare professionals from four nursing homes (41 per group).	Common education on empathy/aging in both groups; experimental group additionally used the GERT aging-simulation suit.	Self-reported Interpersonal Reactivity Index and clinical-empathy-related outcomes after training.	No between-group difference in IRI empathy scores; the simulation group showed improvement in selected secondary perceptions/experience measures.	This tests the incremental effect of simulation over education, not education versus no intervention. Confirm publication status and complete variance data before meta-analysis.

Table S3. Study-level evidence on compassion, caring, and humanistic practice

Study	Setting, design, and sample	Intervention and comparator	Outcome, source, and timing	Findings reported in the PDF	Quantitative-synthesis implications
Tai and Lin (2026)	Taiwan; ward-clustered quasi-experimental pretest–posttest study; eight wards; 60 intervention and 61 control nurses.	Structured experiential psychological-support communication training versus no training.	Nurse knowledge, attitudes, skills, confidence, and job stress at baseline, 3, and 6 months; ward-aggregate patient satisfaction and quality of life.	Most nurse competencies showed favorable group×time interactions. Ward-level patient satisfaction and quality of life favored intervention wards at 3 months but were not sustained at 6 months.	Patient outcomes are ward aggregates and cannot be linked to individual nurses. Use adjusted mixed-model effects and keep nurse and patient-level syntheses separate.
Mau et al. (2025)	Indonesia; quasi-experimental controlled pre/post study; 120 inpatient nurses (60 per group).	Structured training plus mentoring over 3 months versus control.	Validated questionnaires assessing caring knowledge, attitudes, and actions.	Large improvements favored intervention for knowledge (partial η^2=.882), attitudes (.711), and actions (.737); overall between-group improvement was reported p<.001.	Partial η^2 can be presented but should not be mixed directly with post-test SMDs. Verify allocation and baseline comparability under ROBINS-I.
Park et al. (2021)	South Korea; quasi-experimental controlled study; 88 nurses (experimental 39; control 49).	Patient-centered-care program using patient-role enactment and storytelling versus delayed training.	Perceived PCC, compassion, and knowledge transfer before/after training.	Changes in PCC perception (p=.001) and compassion (p=.006) were greater in the intervention group; knowledge transfer correlated with PCC (r=.55) and compassion (r=.63).	Compassion scale may support SMD if complete group data are reported. Correlations are secondary and not causal intervention estimates.
Hussien et al. (2025a)	Saudi Arabia; controlled quasi-experimental study; 100 nurses.	Simulation-based compassionate-care training versus control.	Caregiving behavior, self-efficacy, and compassion competence (communication, sensitivity, insight) before/after training.	Intervention means increased for caregiving behavior (44.2 to 77.32) and self-efficacy (21.16 to 33.72), with significant gains across	Potential SMDs if post-test/change SDs are available. Avoid using within-group change alone as the comparative effect.

Study	Setting, design, and sample	Intervention and comparator	Outcome, source, and timing	Findings reported in the PDF	Quantitative-synthesis implications
				compassion domains; controls changed minimally.	
Antonini et al. (2021)	Switzerland; cluster randomized trial; 101 hemodialysis nurses in 10 hospitals.	Brief Watson Human Caring-based educational intervention versus control.	Caring Nurse–Patient Interaction Scale (EIIP-70) and quality of working life at four time points.	The intervention reinforced several humanistic caring attitudes/behaviors, although effects varied across EIIP-70 dimensions and time; work-life outcomes were secondary.	Strong candidate for domain-specific meta-analysis if EIIP-70 subscales align with other caring studies. Adjust for clustering and repeated measures.
Zhuang et al. (2025)	China; randomized controlled trial; 77 newly recruited ophthalmic nurses (39 intervention; 38 control).	DIKW-based visual-impairment simulation versus traditional training.	Nurses Humanistic Practice Ability Scale and Professional Identity Scale at baseline and post-intervention.	Post-intervention humanistic-practice ability favored training ($t=3.132$, $p<.05$); professional identity also favored training ($Z=-5.222$, $p<.001$).	Humanistic-practice scale is multidimensional and not interchangeable with compassion/caring scales; narrative synthesis unless conceptually aligned subscales are extractable.
Shimran and Ali (2025)	Iraq; quasi-experimental controlled study; 110 critical-care nurses (55 per group).	Integrated ethics and therapeutic-communication educational program versus no program.	Validated 72-item knowledge questionnaire and 34-item observed/practice tool; pre/post assessment.	The experimental group improved knowledge and practices relative to control (reported $p<.05$).	Outcome is ethical/therapeutic communication practice rather than direct patient-reported caring. ROBINS-I; exact means/SDs required before quantitative synthesis.

Table S4. Study-level evidence on trust, rapport, and therapeutic relationships.

Study	Setting, design, and sample	Intervention and comparator	Outcome, source, and timing	Findings reported in the PDF	Quantitative-synthesis implications
Dong et al. (2026)	United States; pilot randomized controlled trial; 59 HIV-care providers (29 training; 30 control).	Online training using validation and motivational interviewing to address intersectional stigma and medical mistrust versus no training.	Survey and role-play skills; training-consistent communication; post-training and 6 months.	Skills addressing mistrust improved moderately immediately (d=.54) but not at 6 months (d=−.14); role-play strategies improved slightly (d=.35), especially autonomy support (d=1.26) and checking understanding (d=.66).	Effect sizes are directly usable if their direction and variance basis are verified. Treat post-training and 6-month effects separately.
Thom (2000)	United States; physician-randomized controlled study; 20 physicians (10 per group) with patient surveys before and 6 months after training.	Short workshop targeting seven trust-building behavior categories versus control.	Patient-reported physician behaviors, trust, satisfaction, and utilization.	Intervention physicians improved in 16 of 19 trust-related behaviors, but the study did not demonstrate a clear statistically significant increase in global patient trust.	Patient-level outcomes clustered within physicians. Extract adjusted intervention×time estimates; retain the null global-trust finding.
Meystre et al. (2013)	Switzerland; controlled trial; 56 trained and 57 untrained oncology clinicians assessed in simulated interviews 6 months apart.	Communication-skills training versus no training.	Working Alliance Inventory–Short Revised and RIAS-coded communication.	Working alliance itself did not improve. Positive talk and psychosocial counseling were associated with stronger alliance, whereas negative talk, biomedical information, and patient questions were associated with weaker alliance.	The communication–alliance associations are not randomized intervention effects. Report the null training effect and keep associations narrative.
Norfolk et al. (2009)	United Kingdom; quasi-experimental pilot; 37	Short rapport-development training based on an empathic-	Rapport knowledge, attitudes, confidence,	Training increased rapport-related knowledge and all three affective dimensions;	Proximal professional outcomes; narrative synthesis unless

Study	Setting, design, and sample	Intervention and comparator	Outcome, source, and timing	Findings reported in the PDF	Quantitative-synthesis implications
	general-practice trainees.	skills model versus comparison group.	and behavior dimensions.	behavioral transfer was more limited/variable.	complete controlled change data are available.
Murray et al. (2015)	United Kingdom/Ireland; cluster/clinic-randomized trial; 24 physiotherapists and 24 patients with chronic low back pain.	Eight-hour self-determination-theory communication training versus wait-list control.	Blinded independent rating with the Health Care Climate Questionnaire of need-supportive behavior in an audiotaped clinical session.	Independent ratings strongly favored training (Cohen d=2.27, p<.01).	Usable standardized effect, but small clusters and clinic-level allocation require cautious variance handling.
Smith et al. (1995)	United States; controlled randomized study of internal-medicine/family-practice residents and their patients.	Intensive psychosocial-medicine training versus standard residency education.	Patient satisfaction, perceived empathy/trust-related aspects, and comparison with other physicians.	Patients of trained residents reported more favorable satisfaction and psychosocial-interaction ratings than patients of controls; effects differed across patient-rating dimensions.	Older trial with clustered patients within residents. Extract exact adjusted ANCOVA results from the PDF tables and avoid reconstructing SDs without justification.

Table S5. Outcome-level RoB 2 assessments for individually randomized trials.

Study	Result assessed	D1	D2	D3	D4	D5	Overall	Support for judgment
Beach et al. (2018)	Audio-coded patient-centeredness; post-training	Some concerns	Low	Low	Low	Some concerns	Some concerns	Randomization and allocation concealment were insufficiently detailed; analyses were small and the prespecified selection among several communication outcomes was unclear.
Butow et al. (2008)	Observed cue-eliciting/responding behavior; 6 months	Some concerns	Low	Some concerns	Low	Some concerns	Some concerns	Sequence generation was reported, but concealment, losses at follow-up, and prespecification across numerous coded behaviors were not fully documented.
Canivet et al. (2014)	Observed pain-communication behavior; post-training/3 months	Some concerns	Low	Low	Low	Some concerns	Some concerns	Random assignment and blinded dual coding supported validity; concealment and a prospective analysis plan for multiple behavioral outcomes were not clearly reported.
Du et al. (2025)	Narrative competence/OSCE relational performance; post-rotation	Some concerns	Low	Low	High	Some concerns	High	Allocation procedures were incompletely described and key relational outcomes were partly participant- or teacher-assessed without blinding, making measurement susceptible to knowledge of assignment.
Fallowfield et al. (2002, 2003)	Observed key communication behaviors; 3 and 12 months	Low	Low	Low	Low	Low	Low	Factorial randomization, clearly defined primary communication outcomes, objective consultation coding, and appropriate longitudinal analyses supported a low-risk judgment.
Haskard et al. (2008)	Patient-reported communication/satisfaction; 1 and 6 months	Low	Low	Some concerns	Low	Some concerns	Some concerns	Physicians were randomized and multilevel analyses addressed patient clustering; incomplete follow-up and multiple outcomes/time points created residual concerns.
Kruijver et al. (2001)	Observed affective communication; post-training	Some concerns	Low	Low	Low	Some concerns	Some concerns	Randomized pretest–posttest design and multilevel analysis were appropriate; concealment and prespecified selection among multiple coded outcomes were unclear.
Liang et al. (2025)	CARE/SEGUE/CPPC-AS OSCE ratings; post-training	Some concerns	Low	Low	Some concerns	Some concerns	Some concerns	Random allocation was reported, but concealment and assessor independence were insufficiently detailed; several overlapping post-training scales were analyzed.

Study	Result assessed	D1	D2	D3	D4	D5	Overall	Support for judgment
Liénard et al. (2010)	Observed communication during clinical rounds; follow-up	Some concerns	Low	Some concerns	Low	Some concerns	Some concerns	Waiting-list randomization was reported; workplace encounters were incompletely observed, and multiple communication categories were analyzed without a clearly accessible analysis plan.
Pollak et al. (2016)	Observer-coded MI empathy/spirit; post-intervention	Low	Low	Low	Low	Low	Low	Randomization, prespecified observer-coded motivational-interviewing outcomes, and analyses accounting for clustered encounters supported a low-risk judgment.
Rask et al. (2009)	Observed nurse communication/patient relationship; post-training	Some concerns	Low	Some concerns	Low	Some concerns	Some concerns	Nurses were randomized, but the small professional sample, changing patient samples, missing observations, and multiple outcomes limited confidence.
Razavi et al. (2002)	Emotionally laden words in simulated interviews; 3/6 months	Some concerns	Low	Some concerns	Low	Some concerns	Some concerns	Randomization and content analysis were reported; allocation concealment, incomplete repeated assessments, and outcome-selection procedures were not fully documented.
Riess et al. (2012)	Patient-rated CARE empathy; post-intervention	Some concerns	Low	Some concerns	Low	Some concerns	Some concerns	Randomization and patient-reported assessment reduced self-report bias, but concealment, missing patient ratings, and prespecification across outcomes were not completely documented.
Serrada-Tejeda et al. (2026)	Self-reported IRI clinical empathy; post-intervention	Low	Low	Low	High	Some concerns	High	Randomization and safety monitoring were clearly described; the main empathy outcome was self-reported by unblinded participants and could plausibly be influenced by intervention awareness.
Zhuang et al. (2025)	Self-reported humanistic-practice ability; post-intervention	Some concerns	Low	Low	High	Some concerns	High	Random allocation was reported, but concealment was unclear and the primary humanistic-practice outcome was self-reported by participants aware of assignment.
Dong et al. (2026)	Role-play communication skills; immediate/6 months	Low	Low	Some concerns	Some concerns	Some concerns	Some concerns	Pilot randomization was adequate; attrition at 6 months, limited information on assessor blinding, and multiple role-play/survey contrasts raised concerns.

Study	Result assessed	D1	D2	D3	D4	D5	Overall	Support for judgment
Murray et al. (2015)	Blinded rating of need-supportive behavior; post-training	Low	Low	Low	Low	Low	Low	Randomization, a clearly defined behavioral outcome, blinded independent assessment, complete professional-level outcome data, and the prespecified primary comparison supported a low-risk judgment.
Smith et al. (1995)	Patient-reported psychosocial interaction/satisfaction; follow-up	Some concerns	Low	Some concerns	Low	Some concerns	Some concerns	Residents were randomized, but older reporting provided limited detail on concealment, attrition, and prespecified selection among patient-rating dimensions.
Thom (2000)	Patient-reported trust and trust-building behaviors; 6 months	Low	Low	Some concerns	Low	Some concerns	Some concerns	Matched physician randomization and clustered analysis were appropriate; the small sample, incomplete patient follow-up, and multiple trust-related outcomes raised concerns.

Note. D1 = bias arising from the randomization process; D2 = bias due to deviations from intended interventions (effect of assignment); D3 = bias due to missing outcome data; D4 = bias in measurement of the outcome; D5 = bias in selection of the reported result.

Table S6. Outcome-level RoB 2 assessments for cluster-randomized trials.

Study	Result assessed	D1a	D1b	D2	D3	D4	D5	Overall	Support for judgment
Gibon et al. (2013); Merckaert et al. (2015)	Observed team communication; simulated and workplace follow-up	Some concerns	Low	Low	Low	Low	Some concerns	Some concerns	Only four radiotherapy teams were randomized, leaving vulnerability to baseline imbalance; recruitment timing and prespecification across many coded outcomes were not fully documented.
Antonini et al. (2021)	Self-reported caring attitudes/behaviors; repeated follow-up	Some concerns	Some concerns	Low	Some concerns	High	Some concerns	High	Ten hospitals were randomized, but post-allocation identification/participation and attrition were possible; the eligible caring outcome was self-reported by nurses aware of allocation.

Note. D1a = bias arising from the cluster randomization process; D1b = bias arising from the timing of identification or recruitment of participants; D2 = deviations from intended interventions; D3 = missing outcome data; D4 = outcome measurement; D5 = selection of the reported result.

Table S7. Outcome-level ROBINS-I assessments for nonrandomized comparative intervention studies.

Study	Result assessed	D1	D2	D3	D4	D5	D6	Overall	Support for judgment
Boissy et al. (2016)	Patient experience after voluntary course participation	Serious	Low	Serious	Moderate	Moderate	Moderate	Serious	Course participation was voluntary and likely associated with motivation, professional role, and institutional characteristics. Although adjusted analyses were reported, residual confounding and self-selection remained plausible.
Caris-Verhallen et al. (2000)	Observer-rated nurse–patient communication after training	Serious	Low	Serious	Moderate	Low	Moderate	Serious	Nonrandom allocation and baseline or contextual differences between nursing units could have influenced communication outcomes. The analyses did not fully address unit-level confounding.
Fisher et al. (2014)	Self-reported relational communication after the intervention	Serious	Low	Serious	Moderate	Serious	Moderate	Serious	Allocation was not demonstrably randomized, the sample was small and selected, and the outcome was reported by participants who were aware of intervention exposure.
Hussien et al. (2025a)	Self-reported empathy after training	Serious	Low	Serious	Moderate	Serious	Moderate	Serious	Quasi-experimental allocation, limited control of confounding, and unblinded professional self-report make the estimated intervention differences vulnerable to bias.
Nixon et al. (2020)	Written empathic responses and confidence after training and at 3 months	Serious	Low	Moderate	Moderate	Serious	Moderate	Serious	Only part of the sample was randomized. Group formation may have introduced systematic differences, and self-reported confidence was susceptible to knowledge of intervention assignment.
Hussien et al. (2025b)	Self-reported compassion competence and caring behavior after training	Serious	Low	Serious	Moderate	Serious	Moderate	Serious	Nonrandom allocation and insufficient confounding adjustment were combined with participant-reported outcomes susceptible to expectancy and social-desirability effects.
Mau et al. (2025)	Self-reported caring actions after 3-month mentoring	Serious	Low	Serious	Moderate	Serious	Moderate	Serious	Purposive recruitment and nonrandom allocation created substantial potential for baseline and motivation-related confounding.

									Outcomes were self-reported by participants aware of intervention exposure.
Park et al. (2021)	Self-reported person-centered care and compassion after training	Serious	Low	Serious	Moderate	Serious	Moderate	Serious	The nonrandom control group may have differed systematically from the intervention group. Limited adjustment and unblinded self-report increased susceptibility to confounding and measurement bias.
Shimran and Ali (2025)	Observed therapeutic communication at 1 month	Serious	Low	Serious	Moderate	Moderate	Moderate	Serious	The reported allocation procedures did not establish robust randomization or concealment. Setting-related and baseline confounding were insufficiently controlled.
Tai and Lin (2026)	Nursing skills and ward-level patient satisfaction at 3 and 6 months	Serious	Low	Serious	Moderate	Moderate	Moderate	Serious	The eight nonrandomized wards were susceptible to organizational, temporal, and patient-mix confounding. Ward-level aggregation further limited attribution of the observed differences.
Meystre et al. (2013)	Working alliance and RIAS-coded behavior at 6 months	Serious	Low	Serious	Moderate	Low	Moderate	Serious	Trained and untrained clinicians were not randomized. Motivation, clinical experience, and workplace characteristics could have confounded alliance and communication outcomes.
Norfolk et al. (2009)	Rapport-related behavior, knowledge, and attitudes after training	Serious	Low	Serious	Moderate	Serious	Moderate	Serious	The quasi-experimental pilot involved small nonrandom groups and limited control of baseline differences. Several principal outcomes were self-reported.

Note. D1 = confounding; D2 = selection of participants; D3 = classification of interventions; D4 = missing datas; D5 = missing data; D6 = selection of the reported result.
